# Supplementary figures and images for: Emerging role of oncogenic ß-catenin in exosome biogenesis as a driver of immune escape in hepatocellular carcinoma
Source: eLife. 2024 Jul 15;13:RP95191. doi: 10.7554/eLife.95191 (PMC11249736; doi:10.7554/eLife.95191)

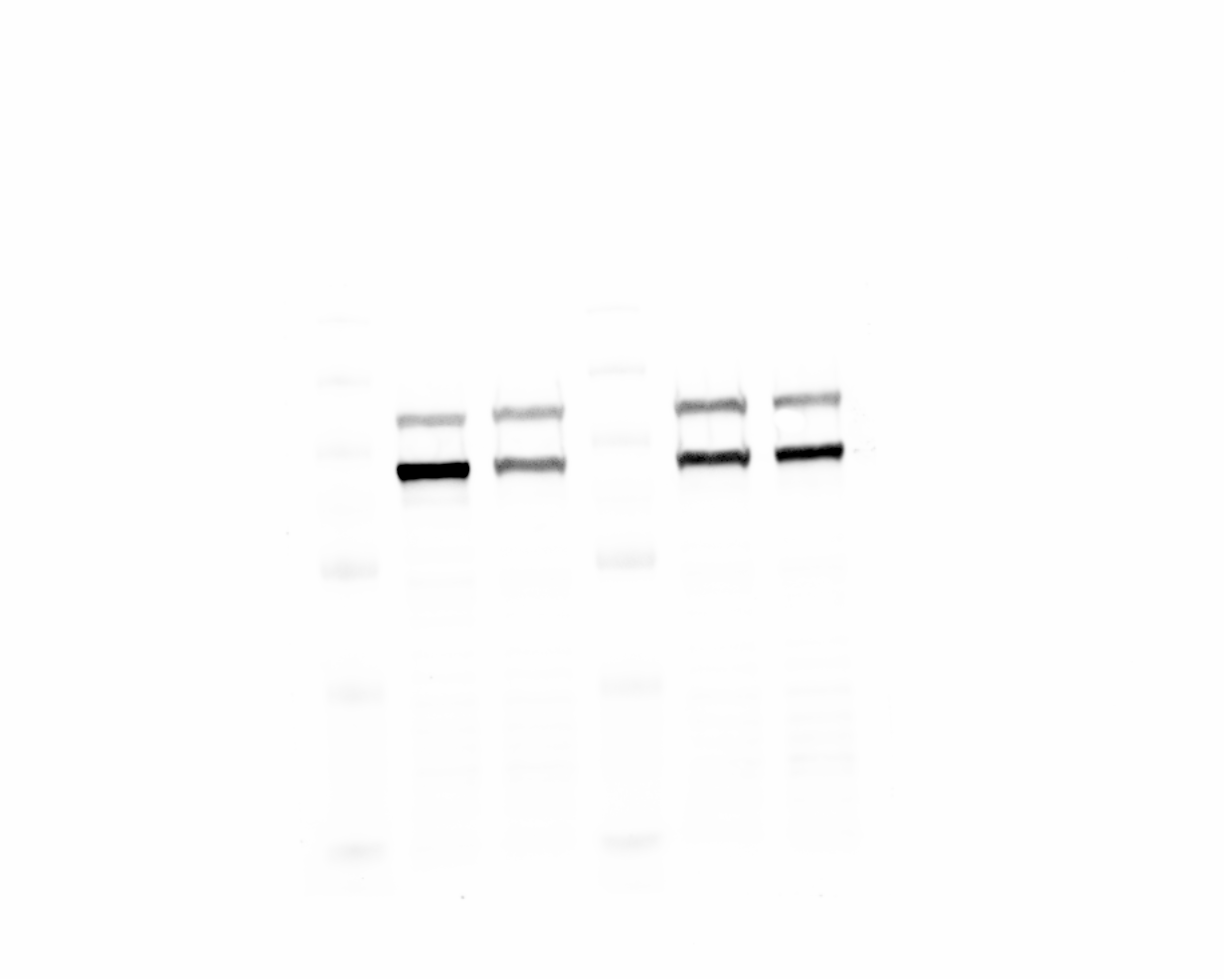

Supplement: Figure 2—source data 1. [file elife-95191-fig2-data1.zip › Figure 2-source data 1/B-catenin.tif]

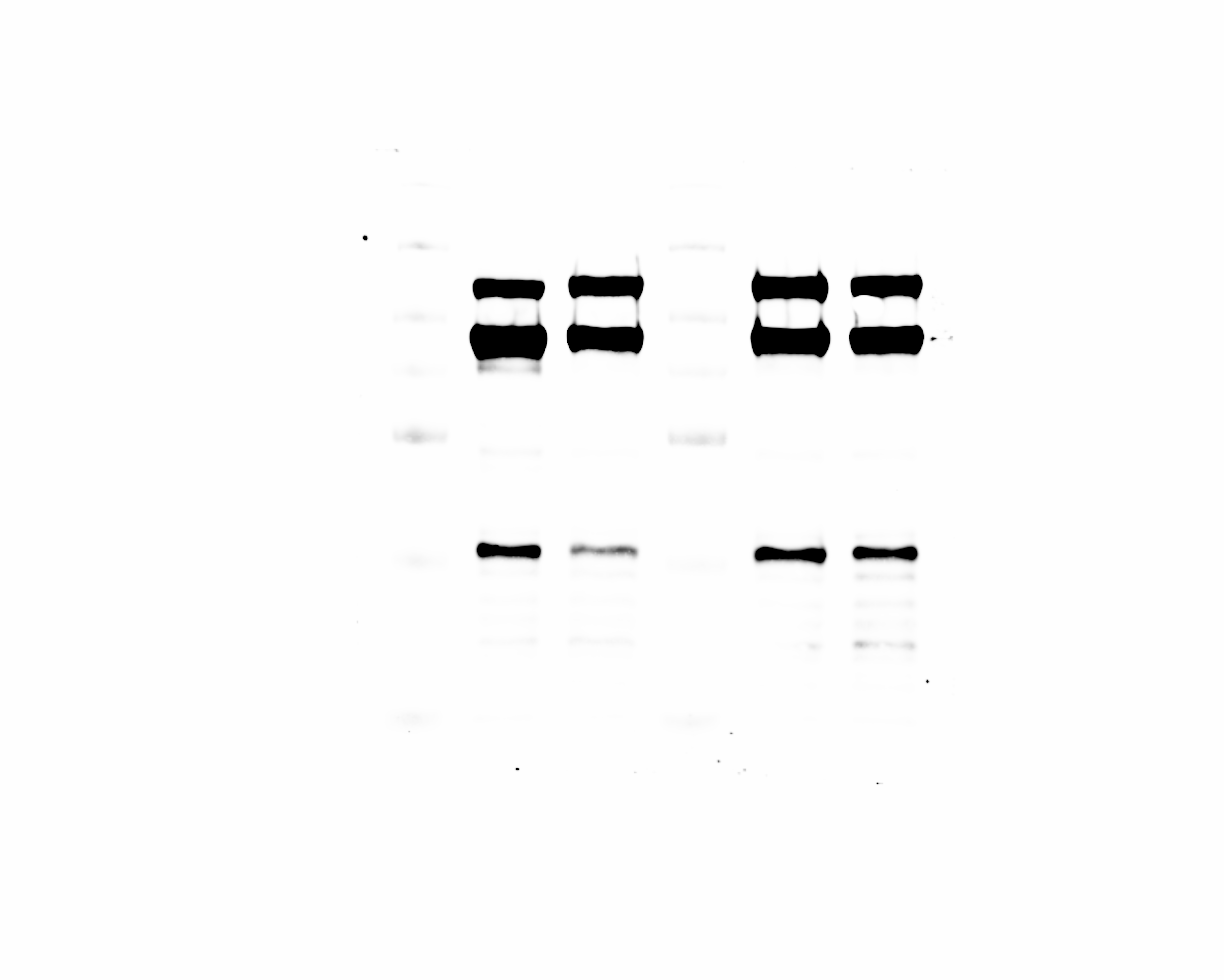

Supplement: Figure 2—source data 2. [file elife-95191-fig2-data2.zip › Figure 2-source data 2/CyclinD1.tif]

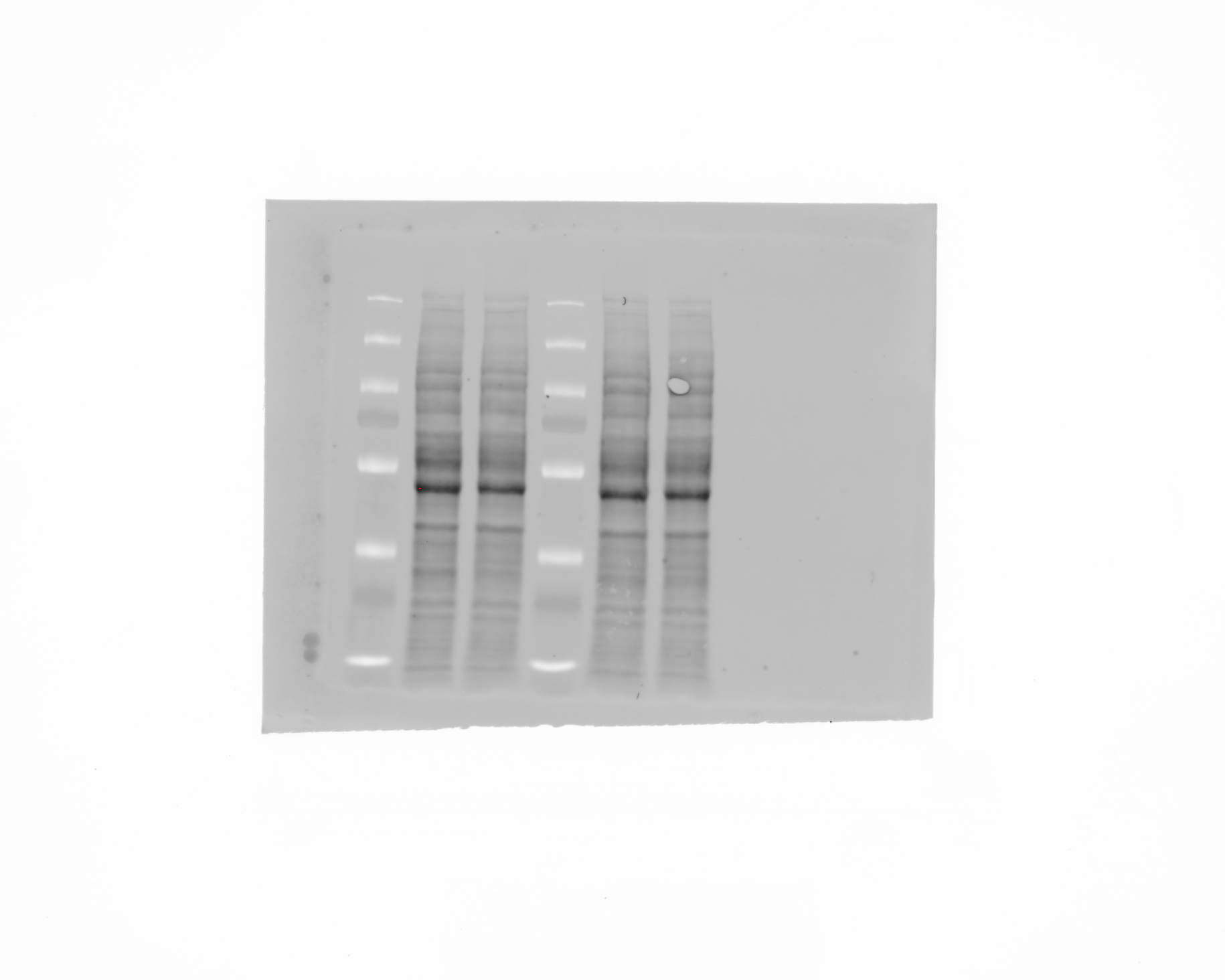

Supplement: Figure 2—source data 3. [file elife-95191-fig2-data3.zip › Figure 2-source data 3/Stain free.tif]

**Figure 2**

**a**

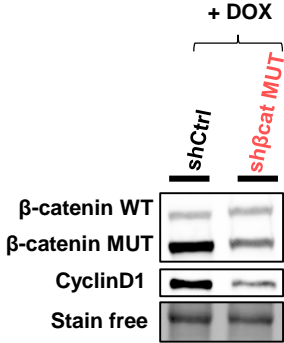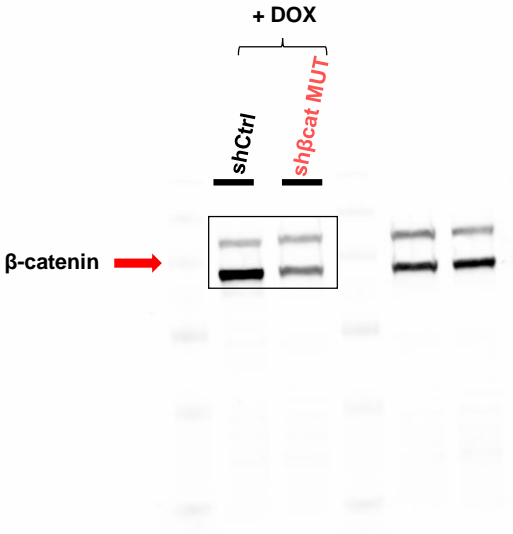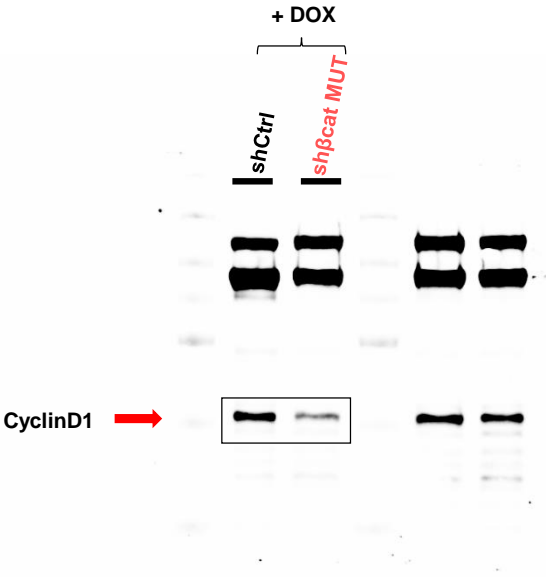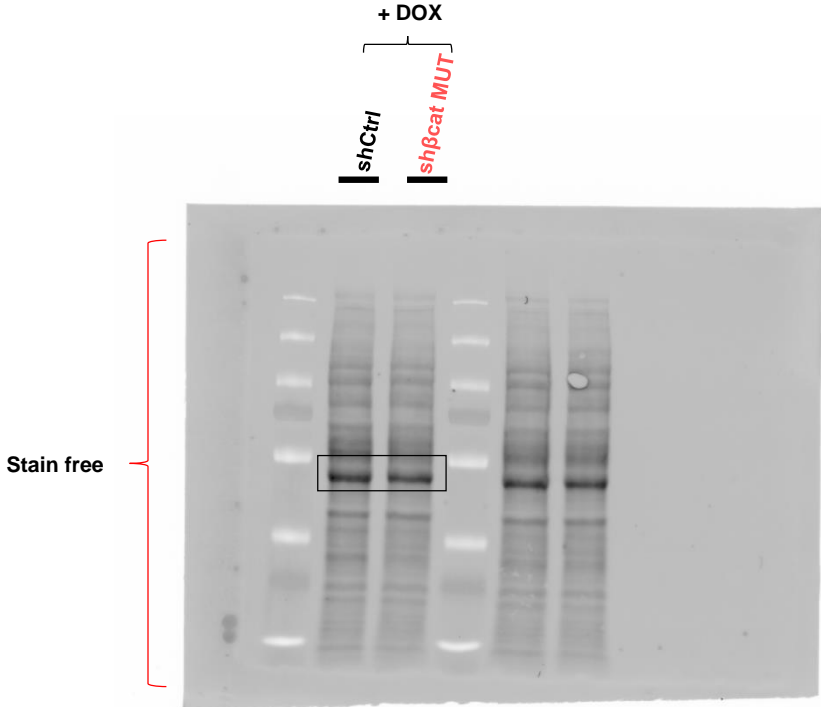

Supplement: Figure 2—source data 4. [file elife-95191-fig2-data4.pdf]

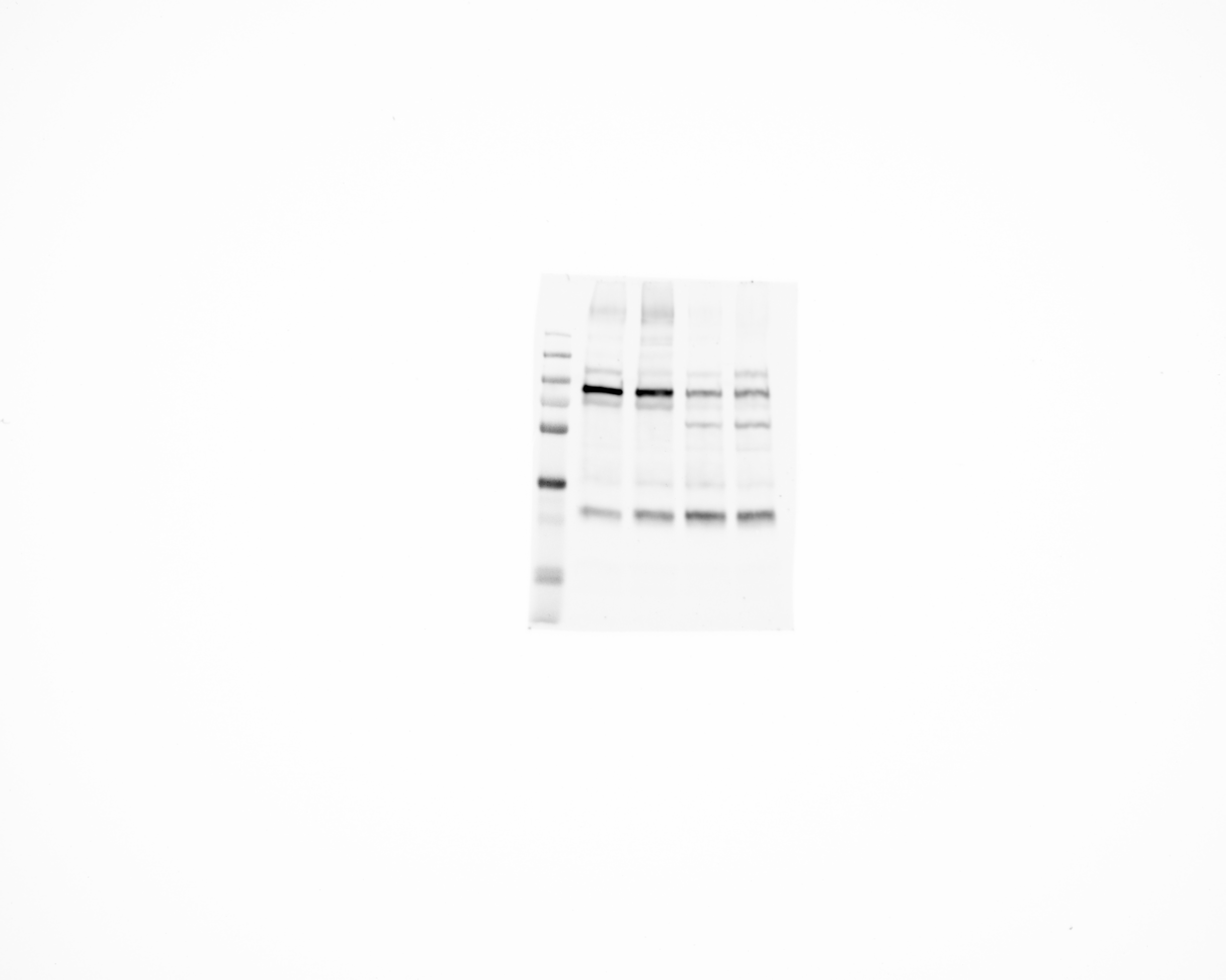

Supplement: Figure 2—source data 5. [file elife-95191-fig2-data5.zip › Figure 2-source data 5/b-catenin.tif]

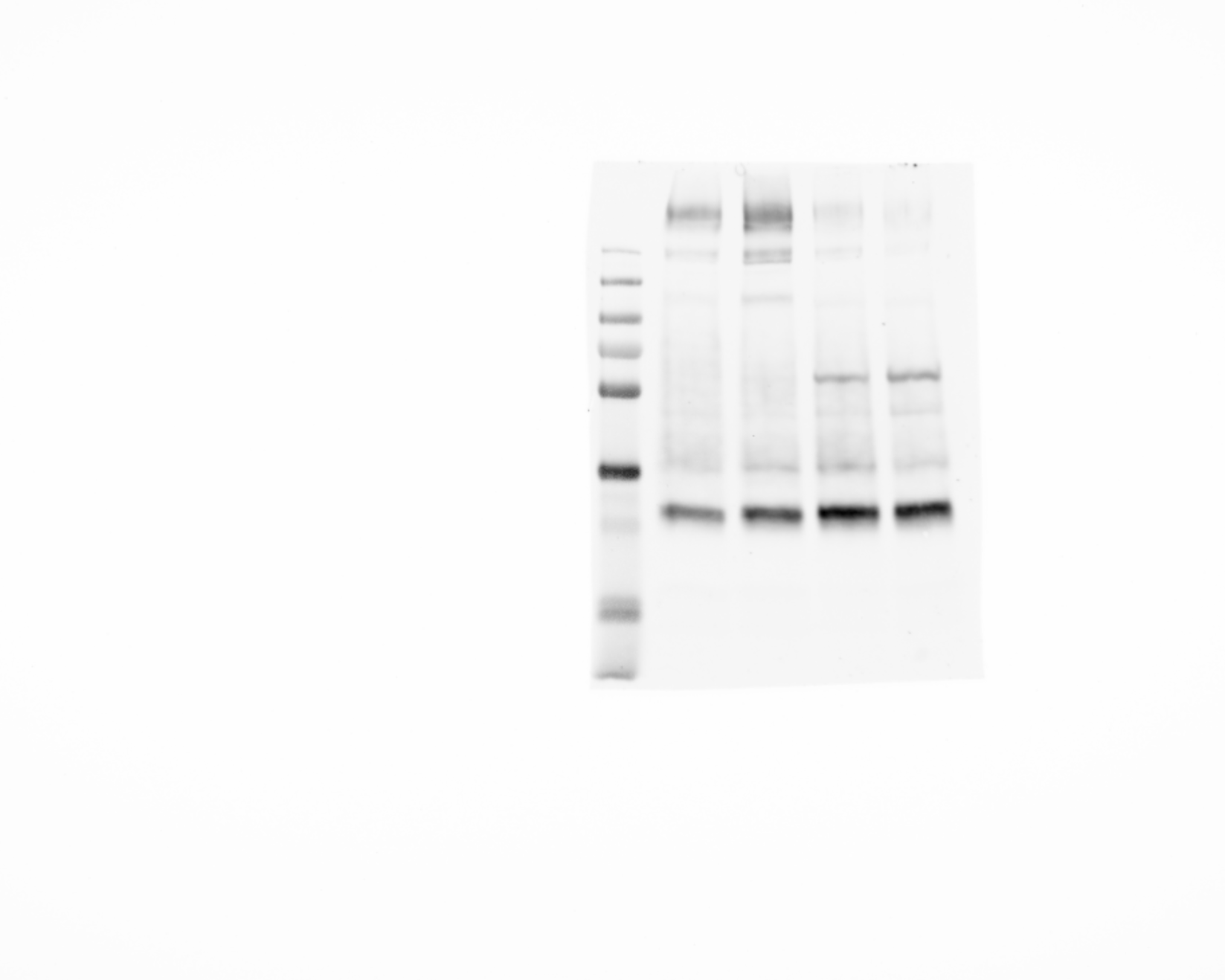

Supplement: Figure 2—source data 6. [file elife-95191-fig2-data6.zip › Figure 2-source data 6/cd63.tif]

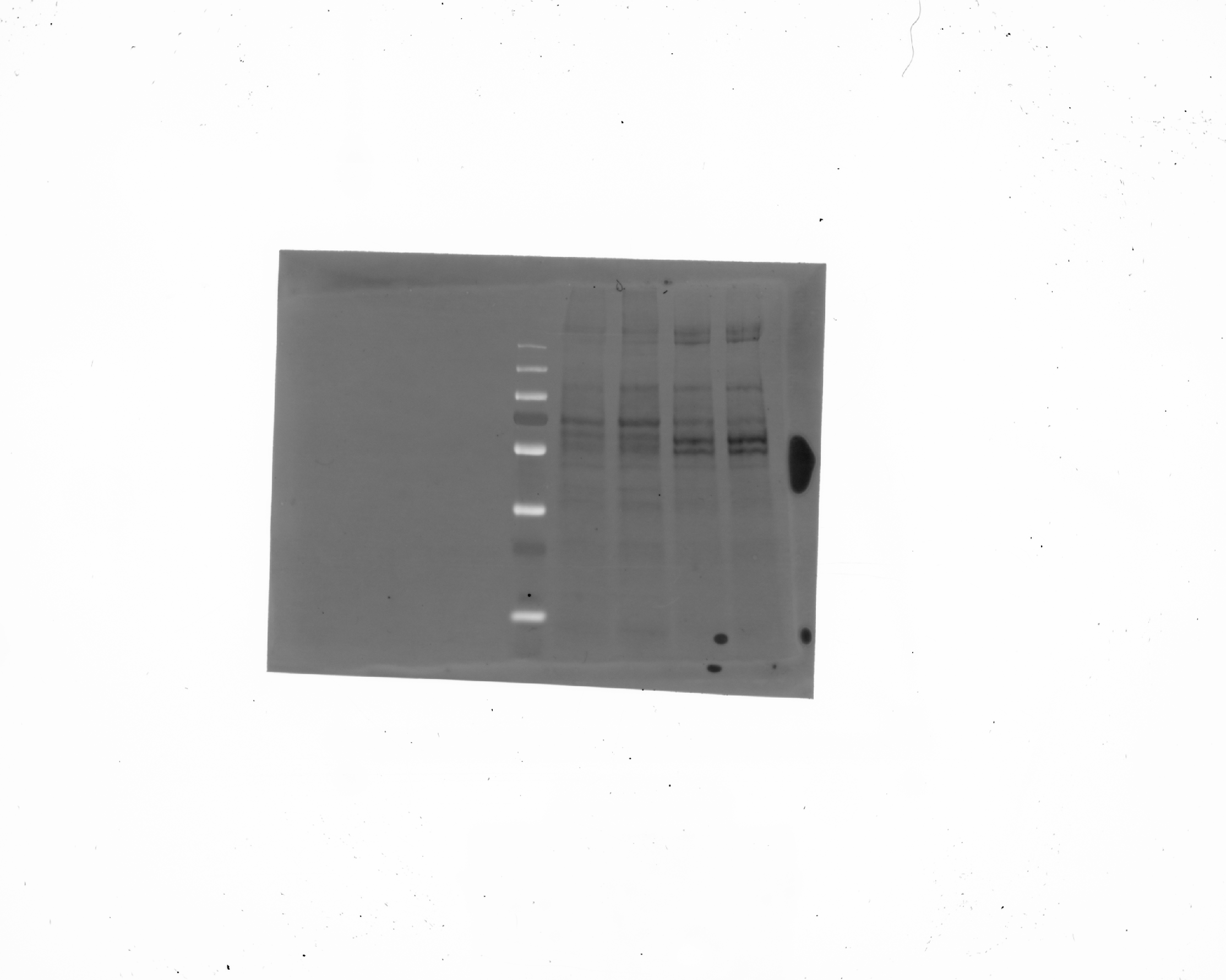

Supplement: Figure 2—source data 7. [file elife-95191-fig2-data7.zip › Figure 2-source data 7/Stain Free.tif]

**Figure 2**

**e**

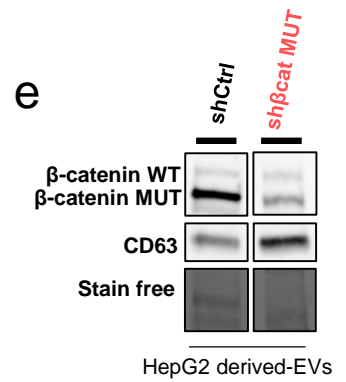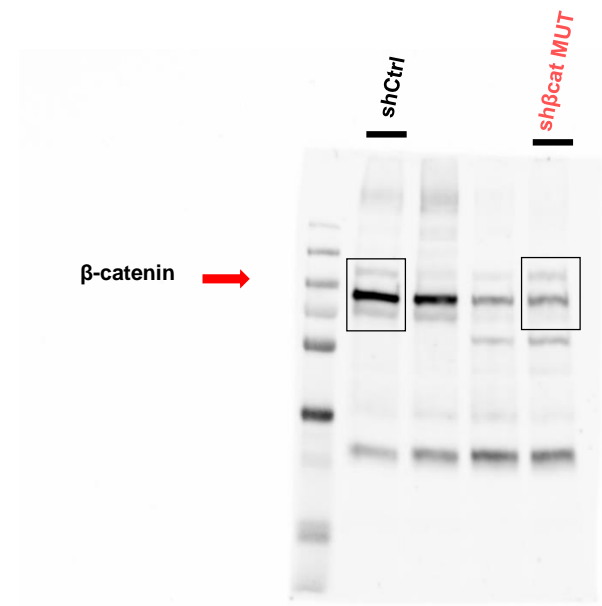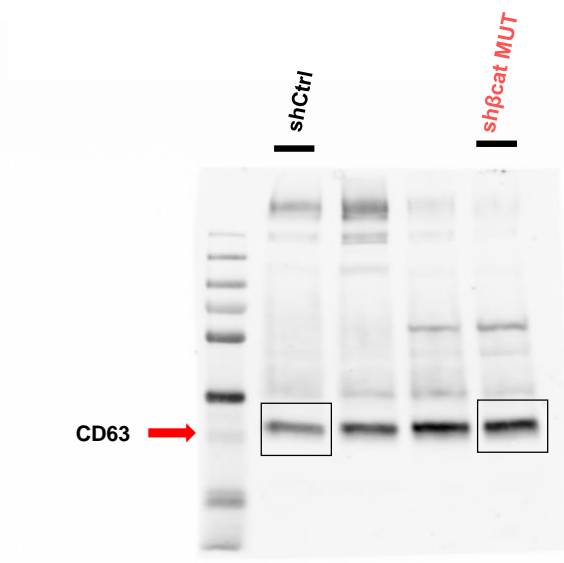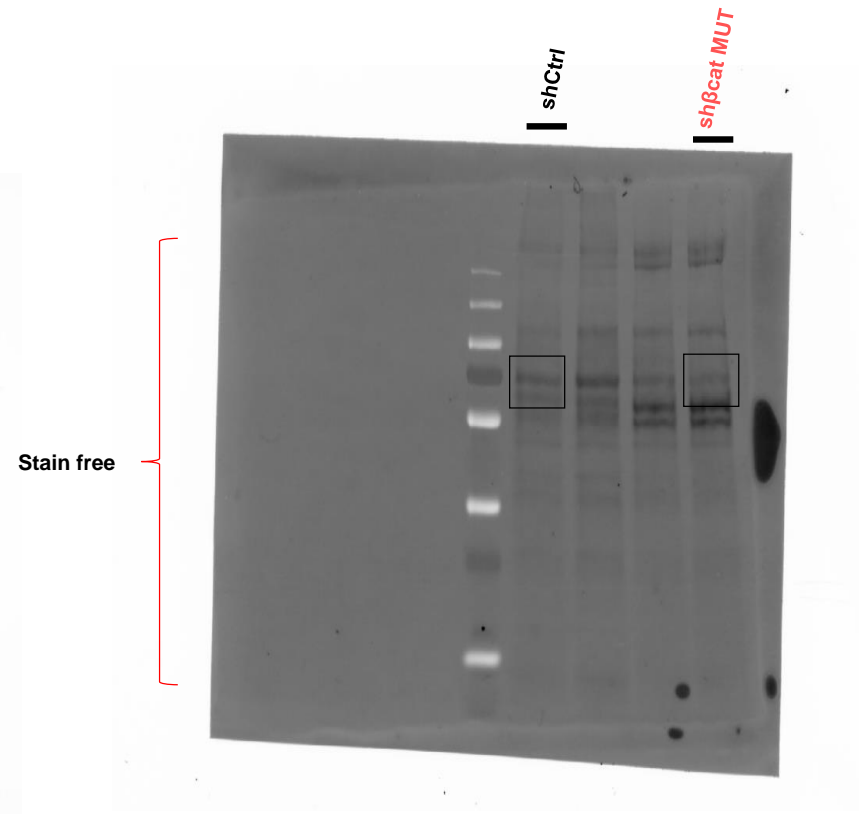

Supplement: Figure 2—source data 8. [file elife-95191-fig2-data8.pdf]

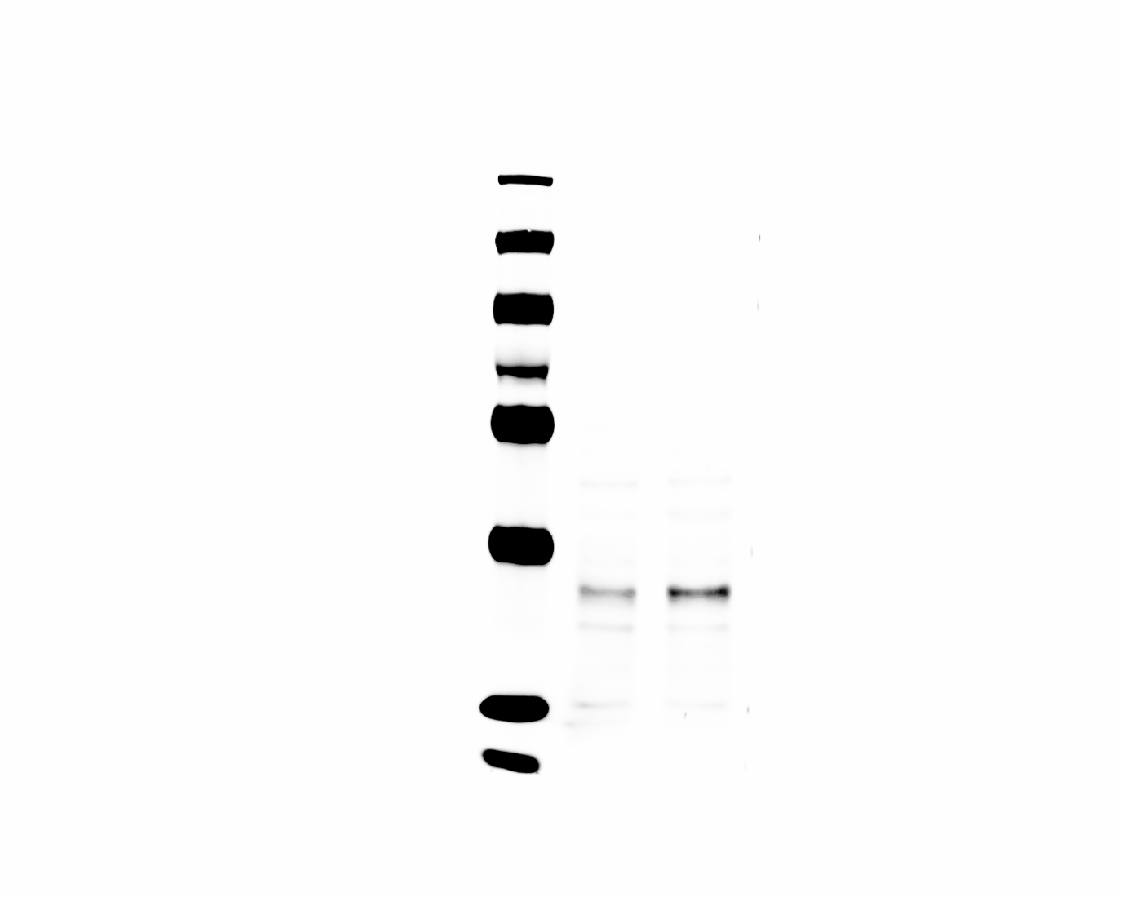

Supplement: Figure 3—source data 1. [file elife-95191-fig3-data1.zip › Figure 3-source data 1/rab27a.tif]

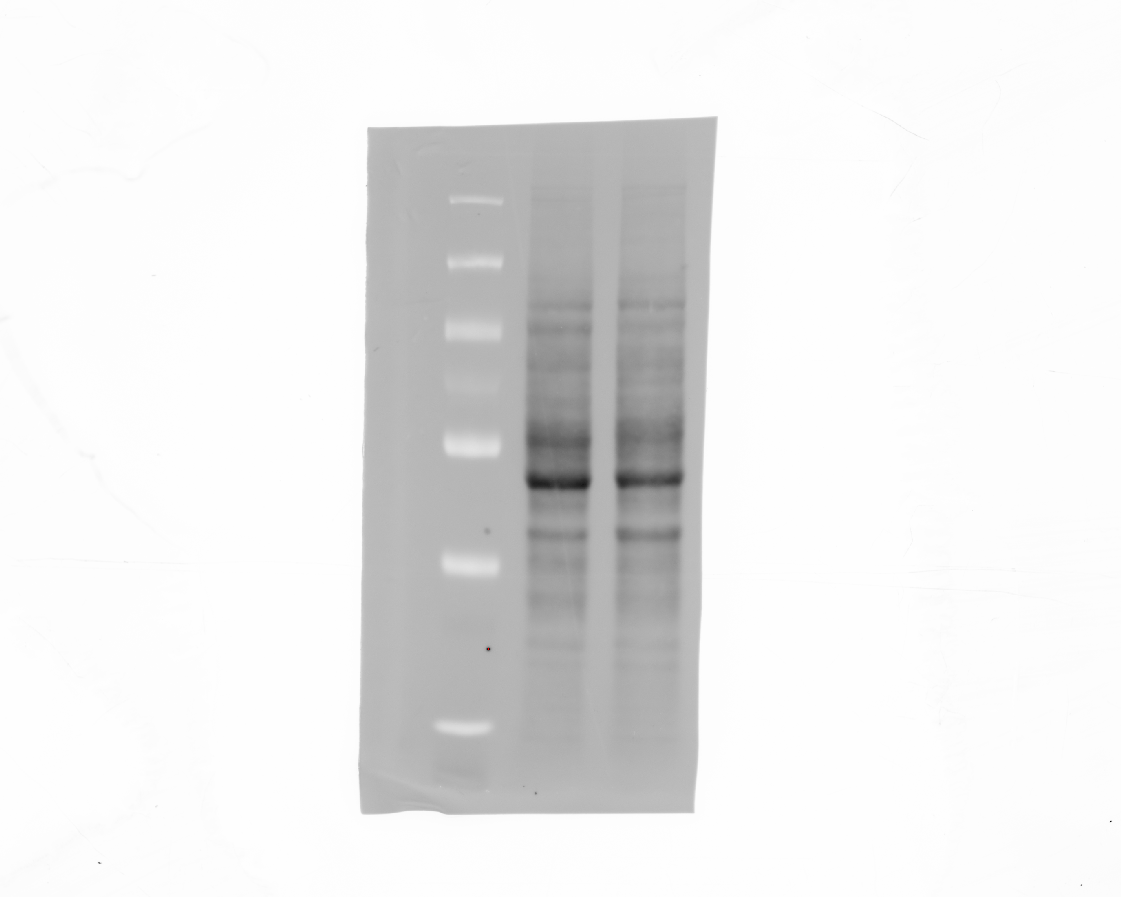

Supplement: Figure 3—source data 2. [file elife-95191-fig3-data2.zip › Figure 3-source data 2/Stain free.tif]

**Figure 3**

**b**

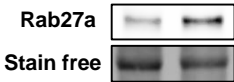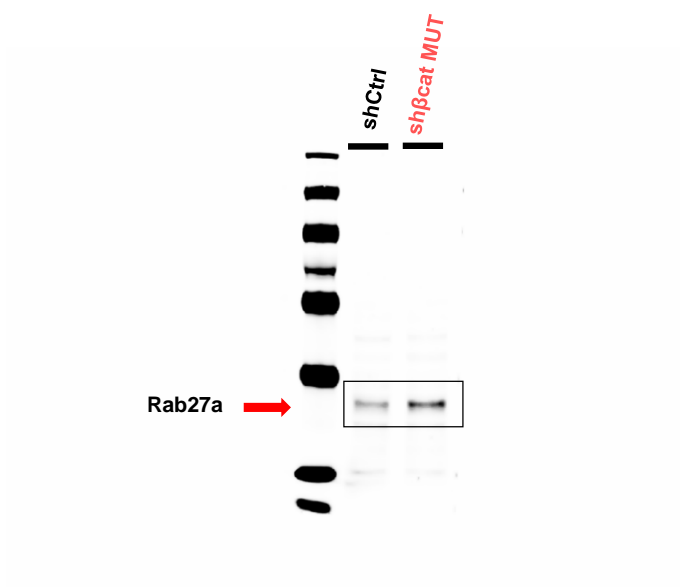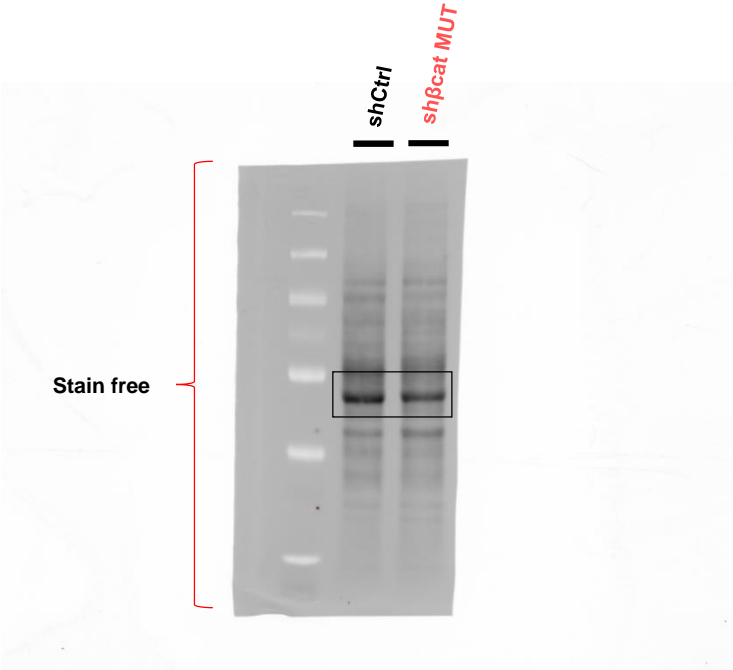

Supplement: Figure 3—source data 3. [file elife-95191-fig3-data3.pdf]

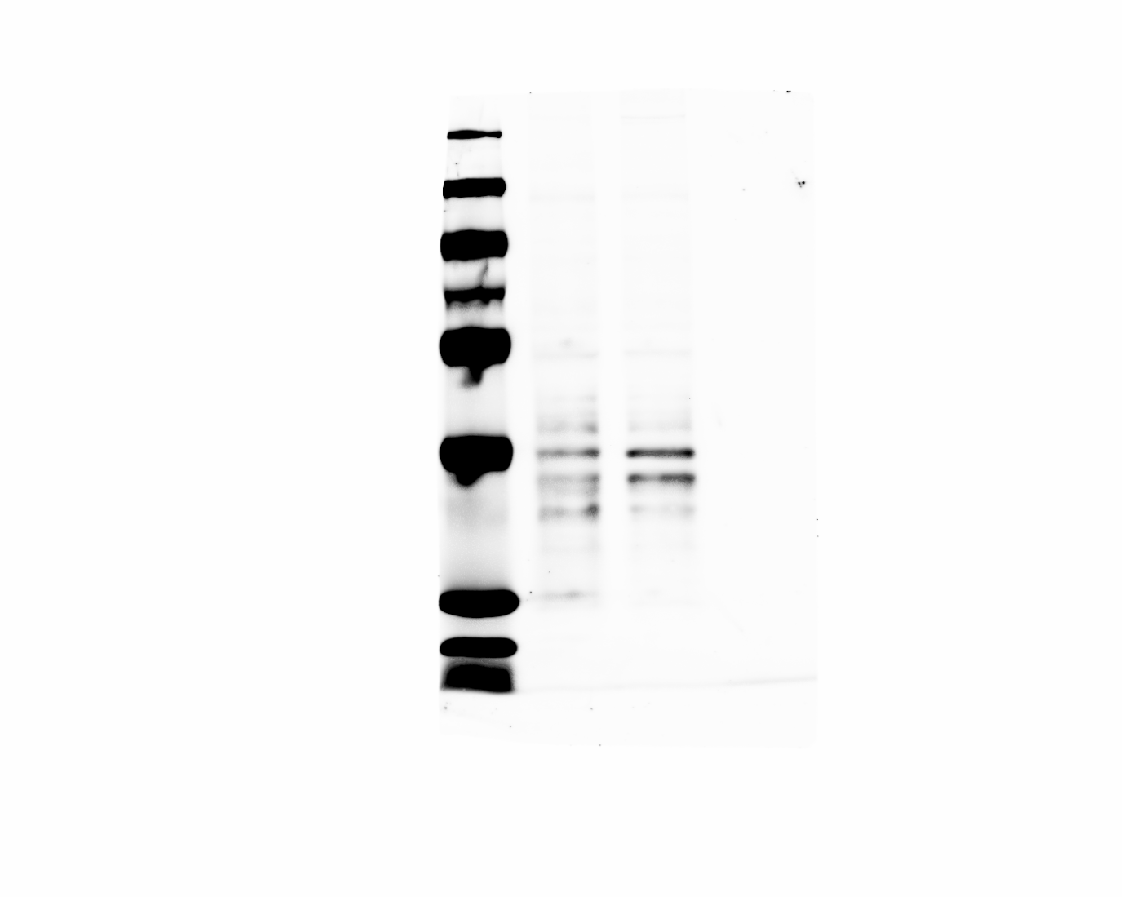

Supplement: Figure 3—source data 4. [file elife-95191-fig3-data4.zip › Figure 3-source data 4/rab27a.tif]

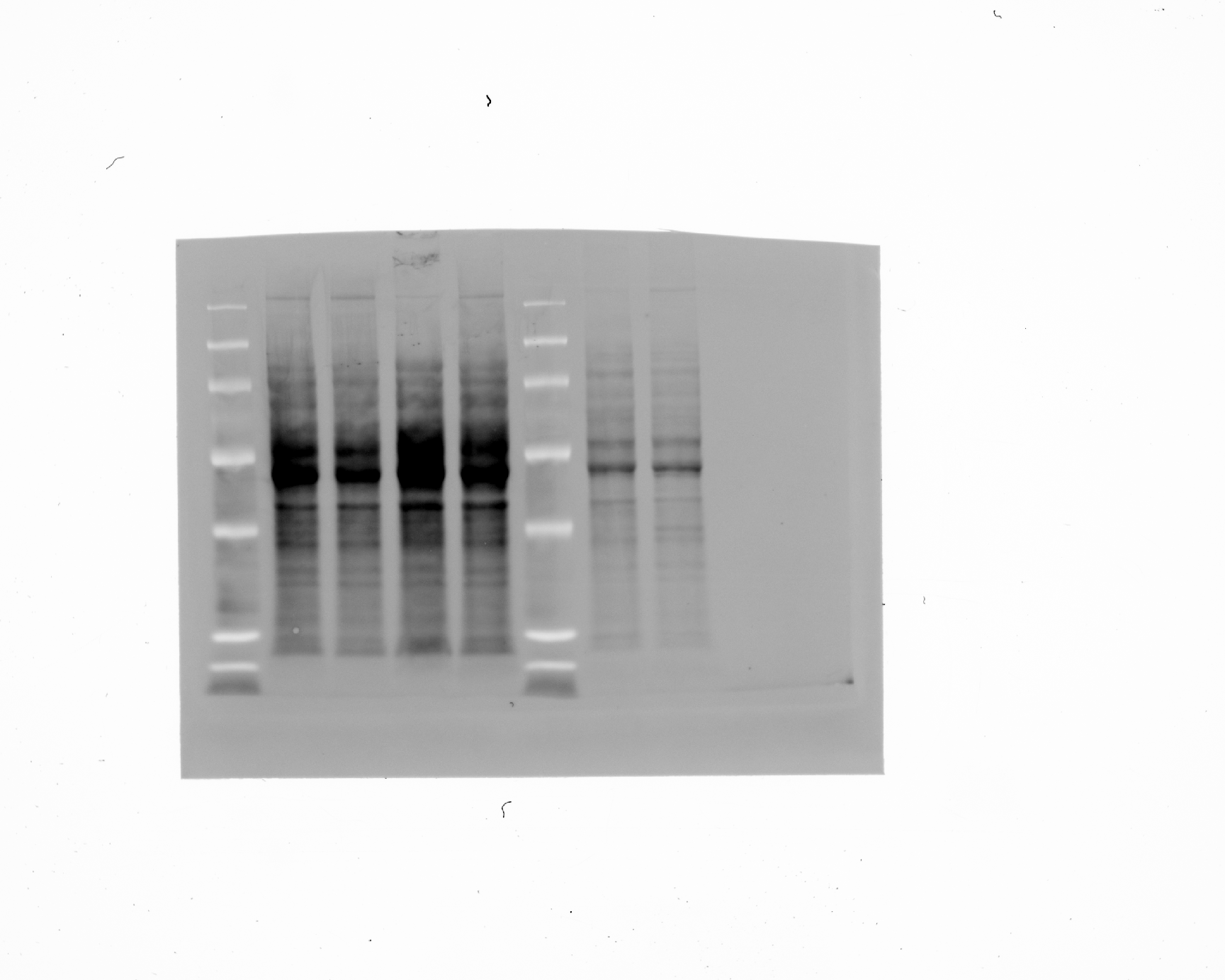

Supplement: Figure 3—source data 5. [file elife-95191-fig3-data5.zip › Figure 3-source data 5/Stain Free.tif]

**Figure 3**

g

Rab27a  
Stain free

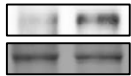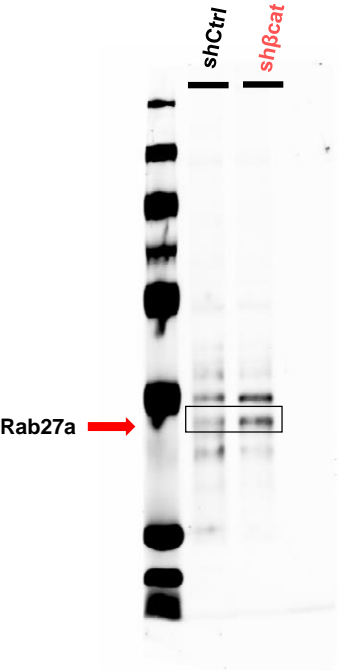

Stain free

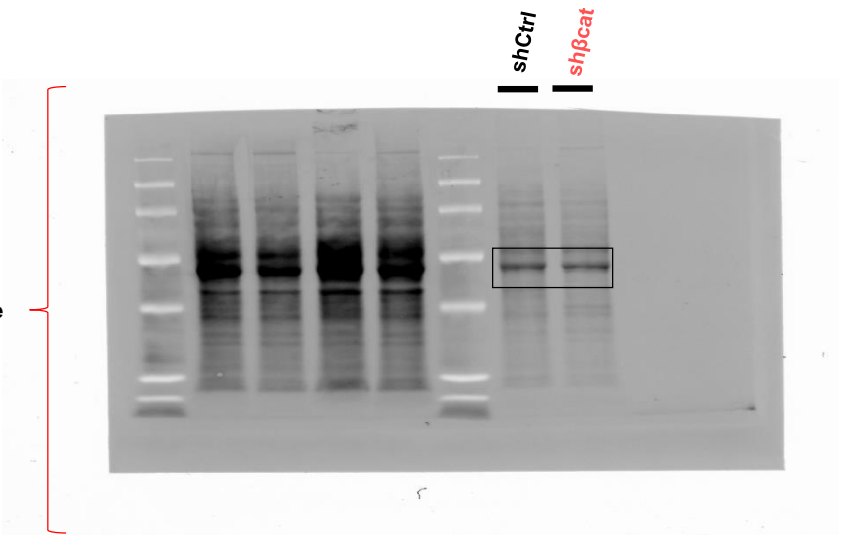

Supplement: Figure 3—source data 6. [file elife-95191-fig3-data6.pdf]

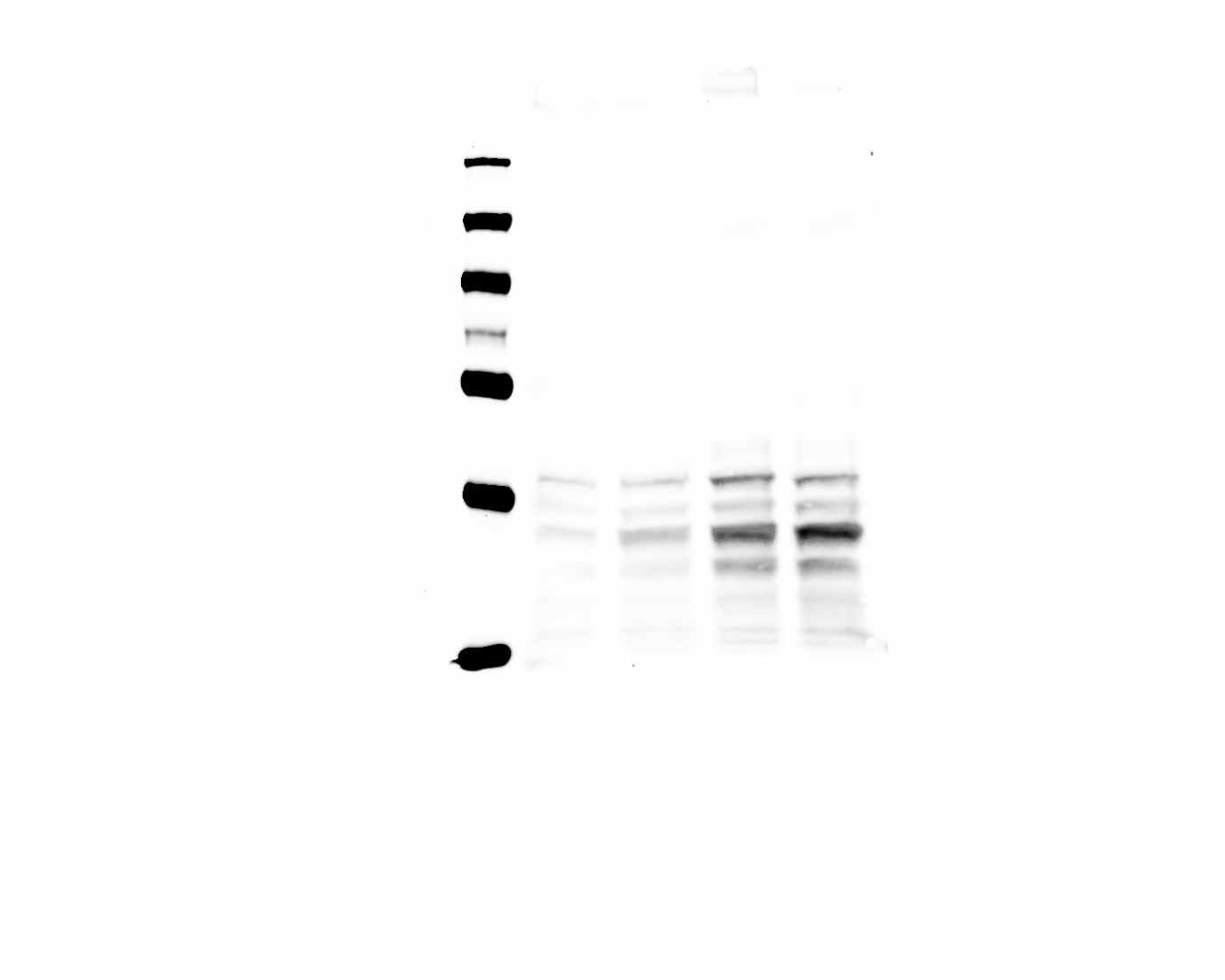

Supplement: Figure 3—source data 7. [file elife-95191-fig3-data7.zip › Figure 3-source data 7/rab27a.tif]

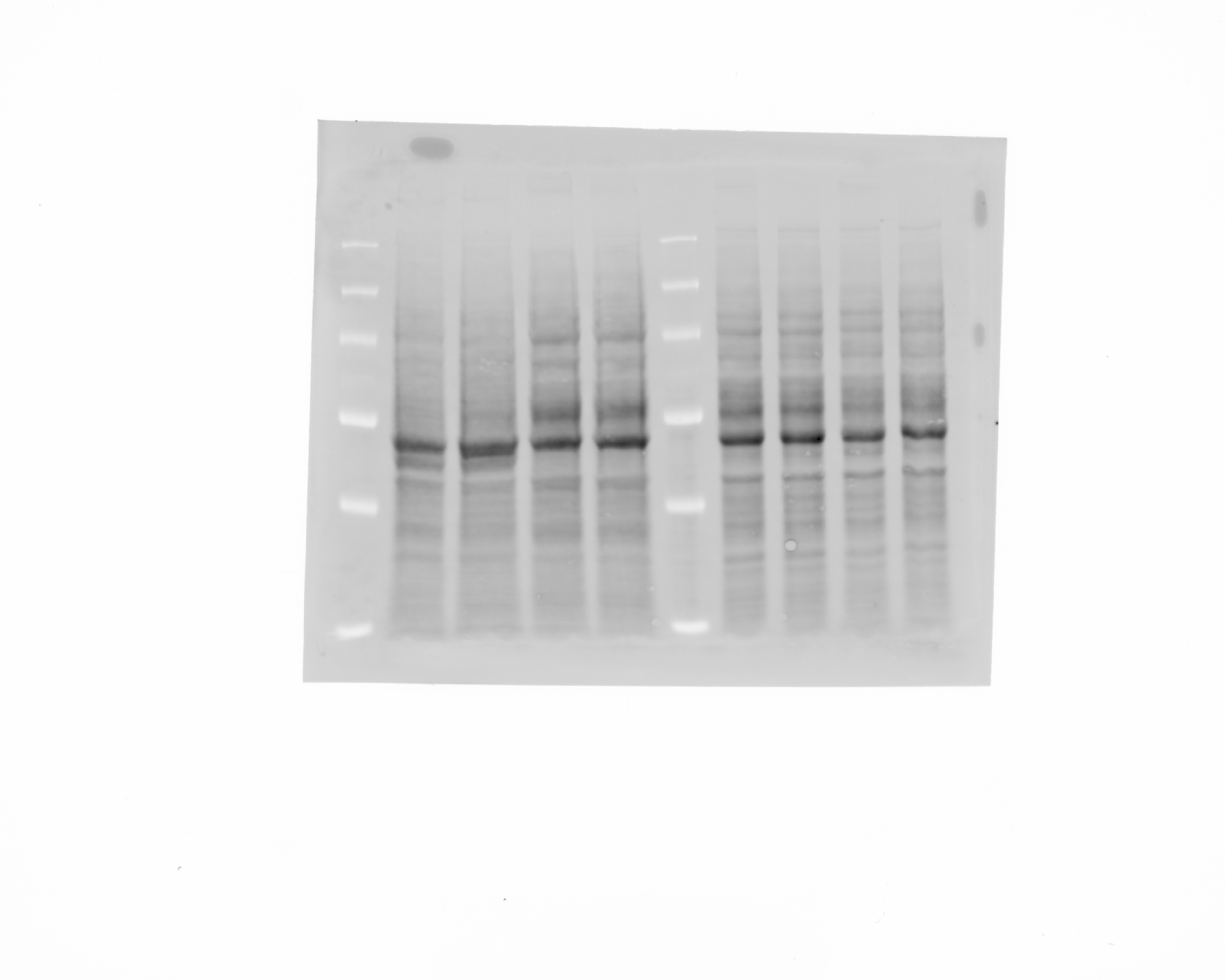

Supplement: Figure 3—source data 8. [file elife-95191-fig3-data8.zip › Figure 3-source data 8/Stain free.tif]

**Figure 3**

h

|            |                                                                                   |
|------------|-----------------------------------------------------------------------------------|
| Rab27a     | 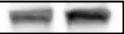 |
| Stain free | 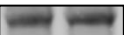 |

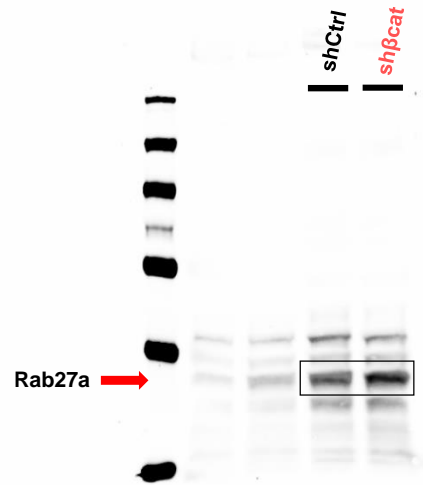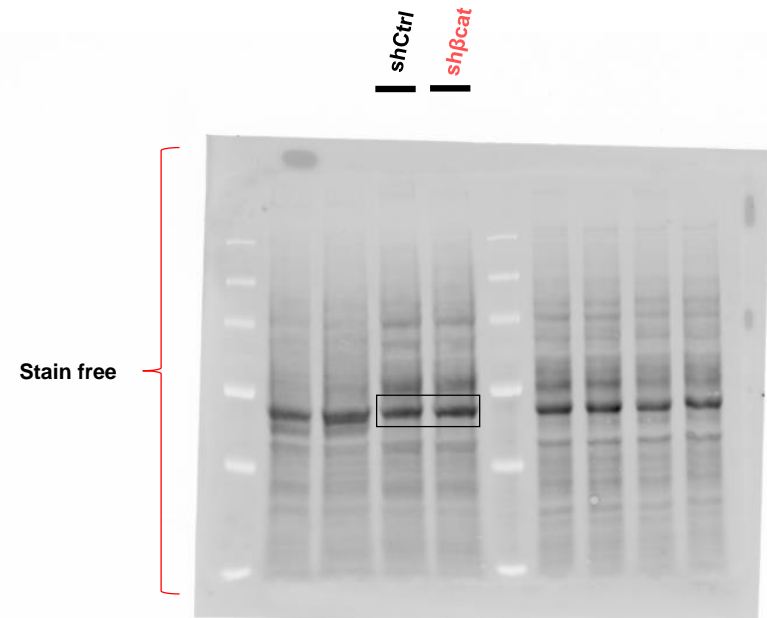

Supplement: Figure 3—source data 9. [file elife-95191-fig3-data9.pdf]

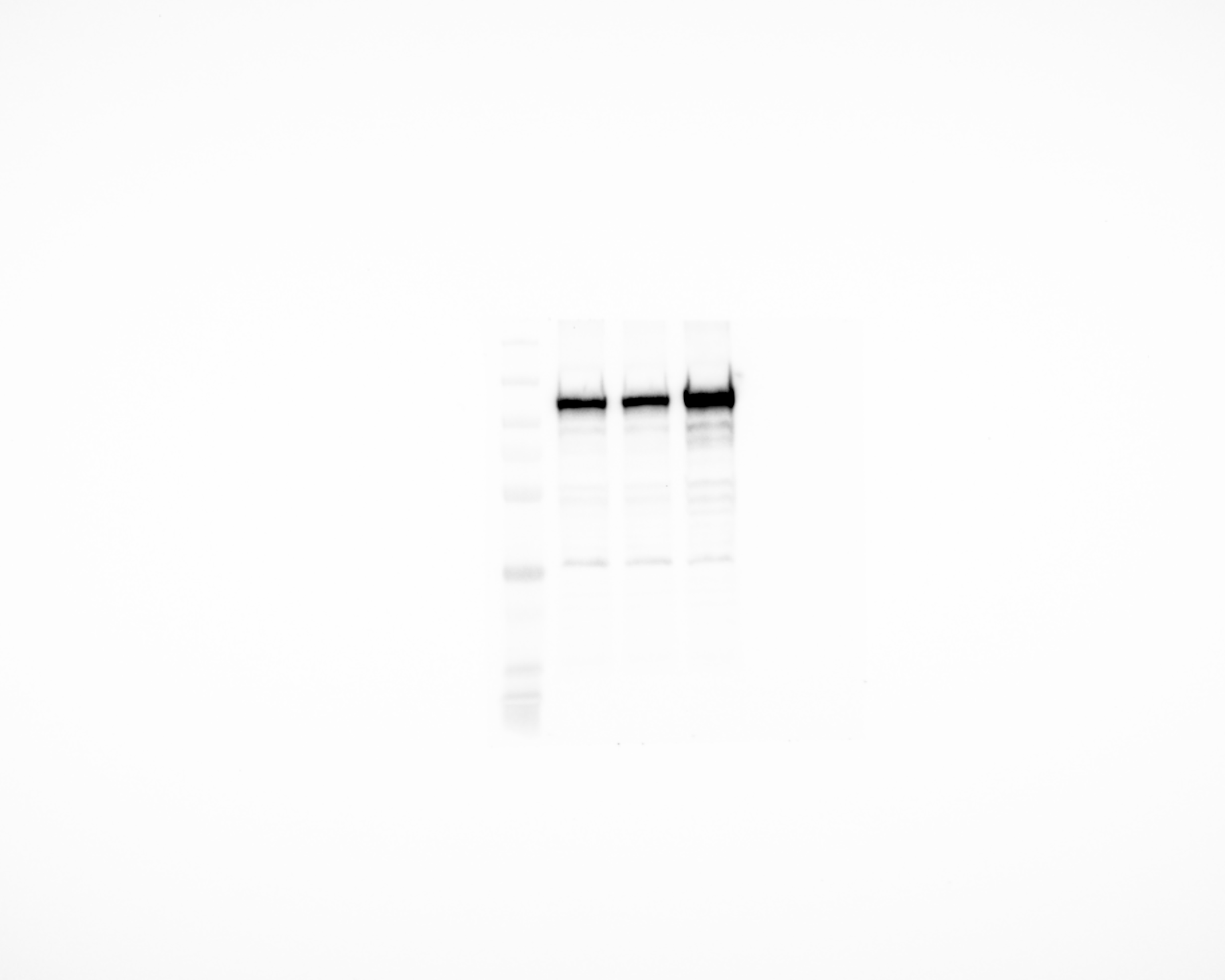

Supplement: Figure 3—source data 10. [file elife-95191-fig3-data10.zip › Figure 3-source data 10/b-catenin.tif]

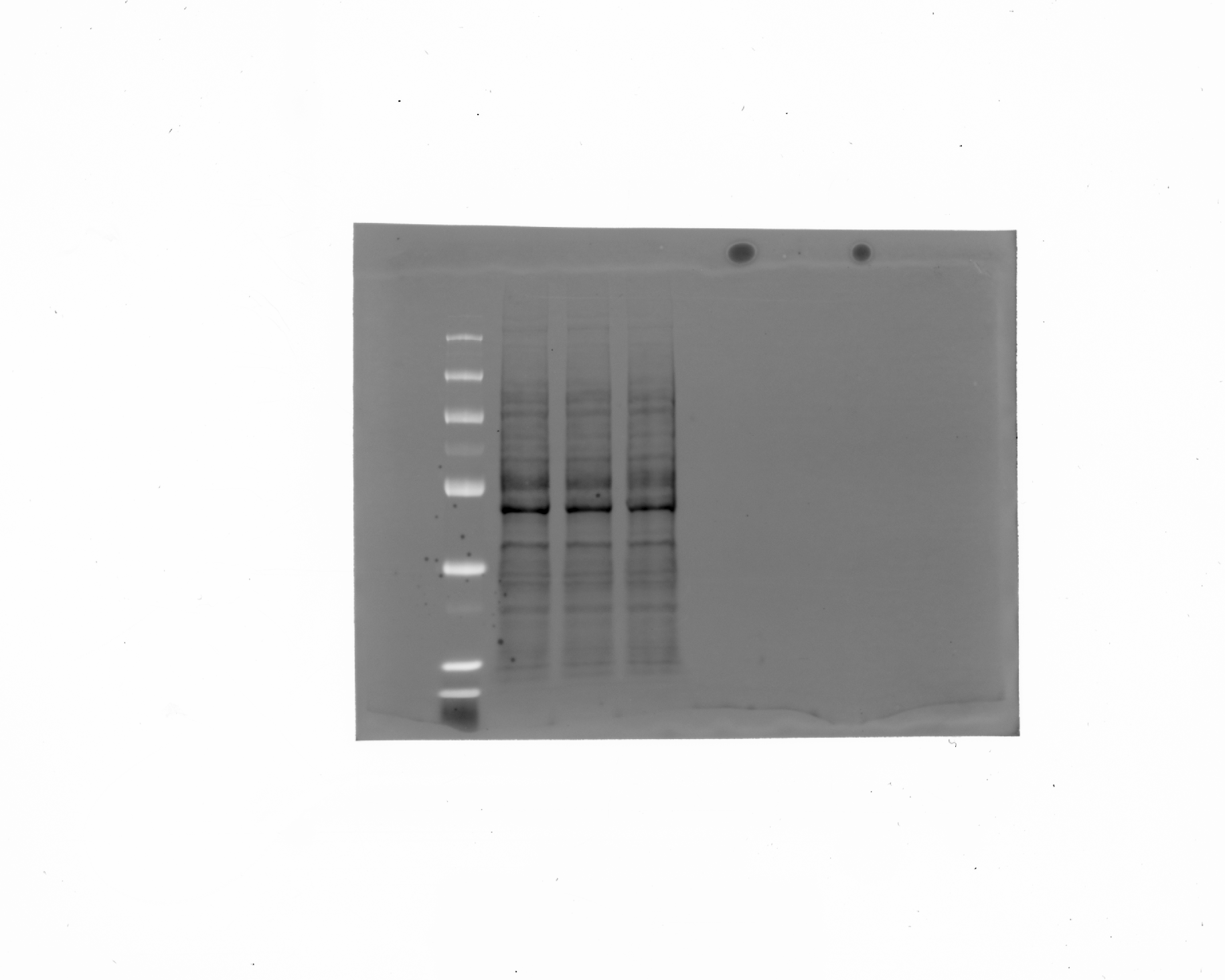

Supplement: Figure 3—source data 11. [file elife-95191-fig3-data11.zip › Figure 3-source data 11/Stain free.tif]

**Figure 3**

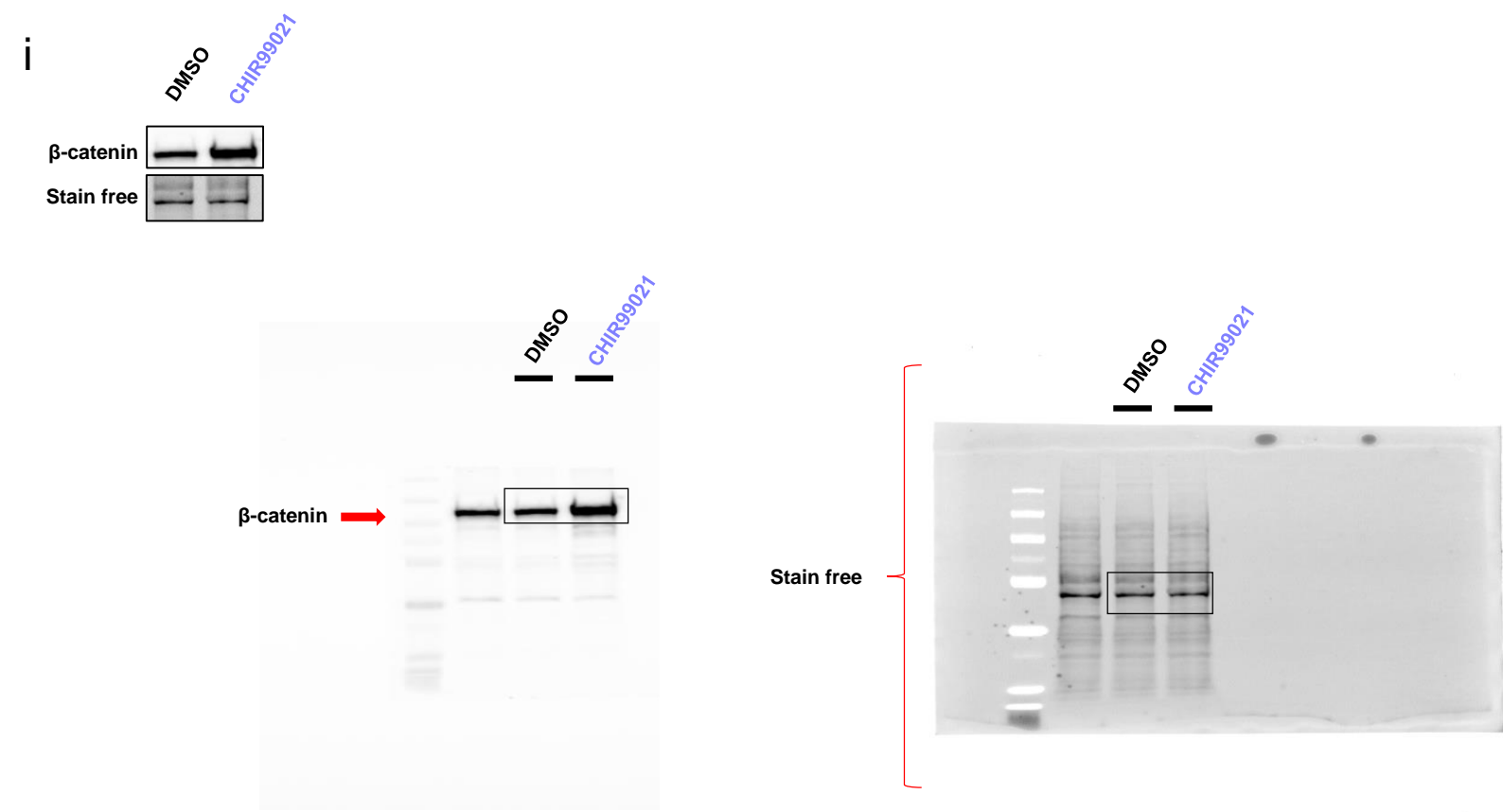

Supplement: Figure 3—source data 12. [file elife-95191-fig3-data12.pdf]

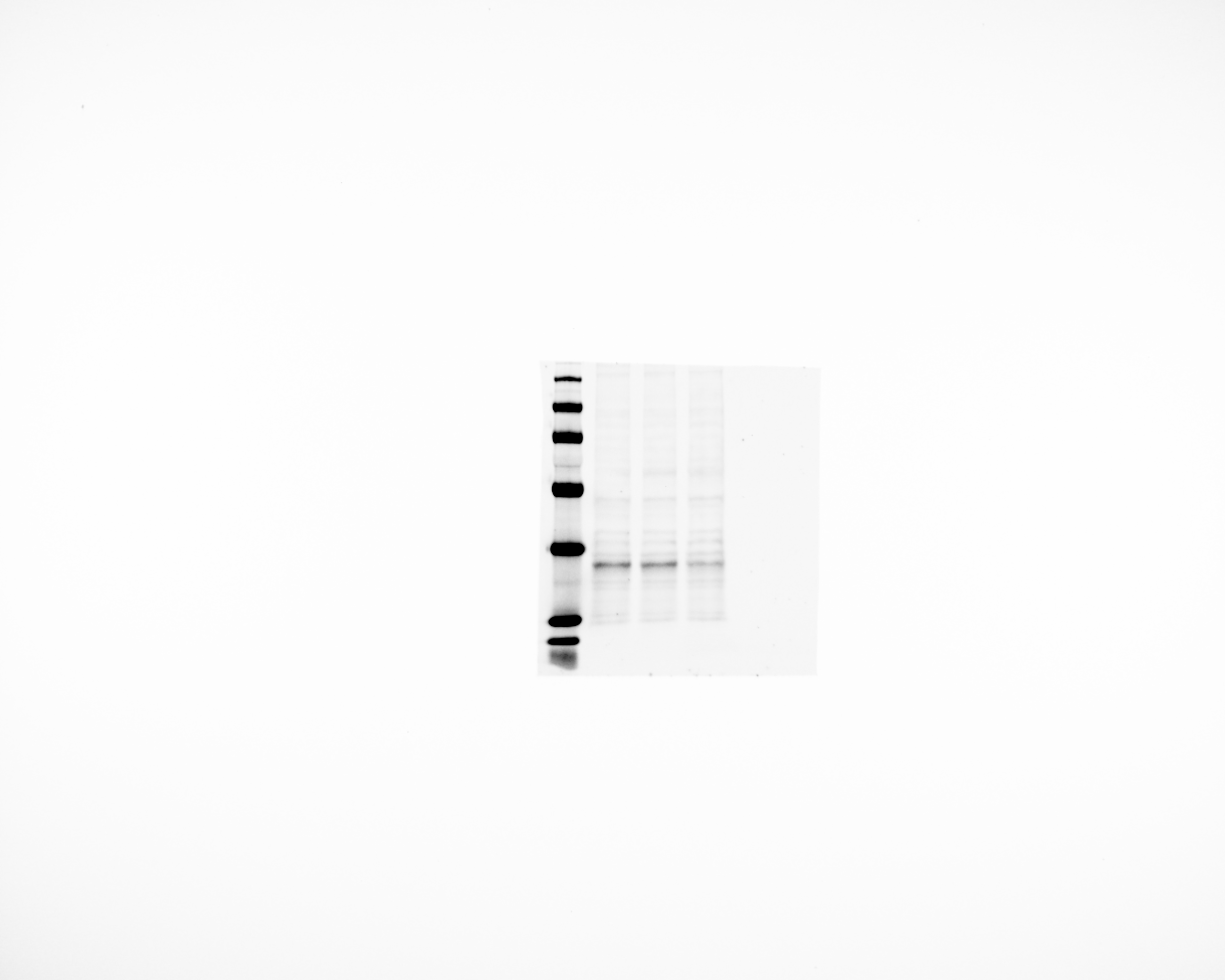

Supplement: Figure 3—source data 13. [file elife-95191-fig3-data13.zip › Figure 3-source data 13/rab27a.tif]

**Figure 3**

Rab27a  
Stain free

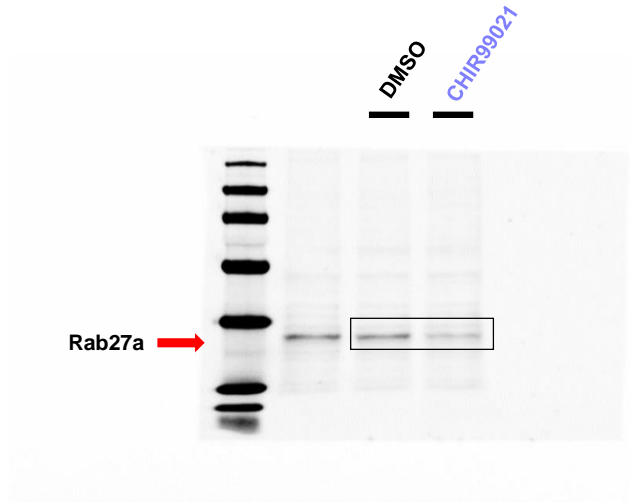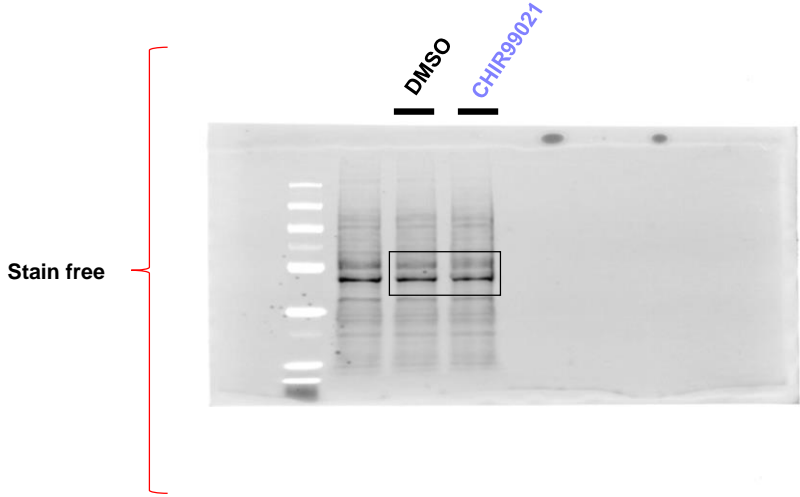

Supplement: Figure 3—source data 15. [file elife-95191-fig3-data15.pdf]

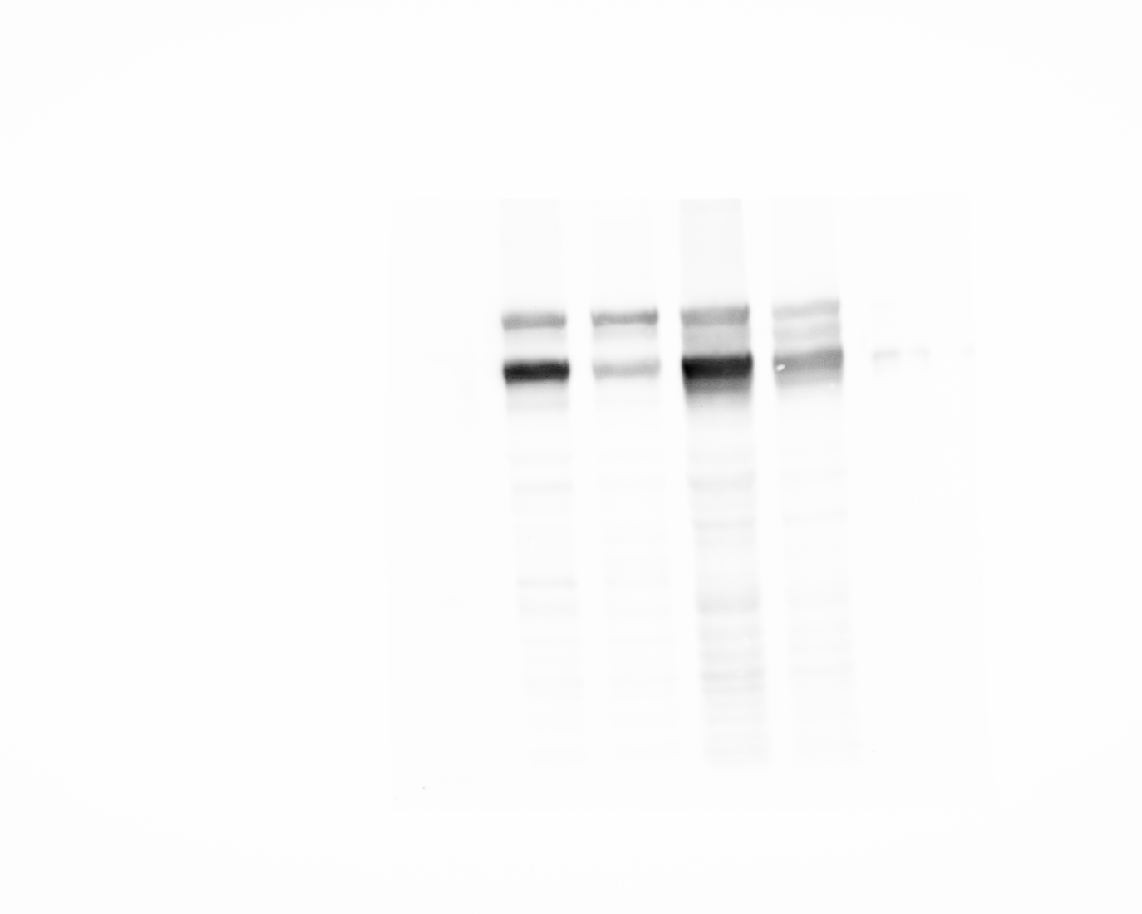

Supplement: Figure 3—figure supplement 1—source data 1. [file elife-95191-fig3-figsupp1-data1.zip › Figure 3-figure supplement 1 source data 1/b-catenin.tif]

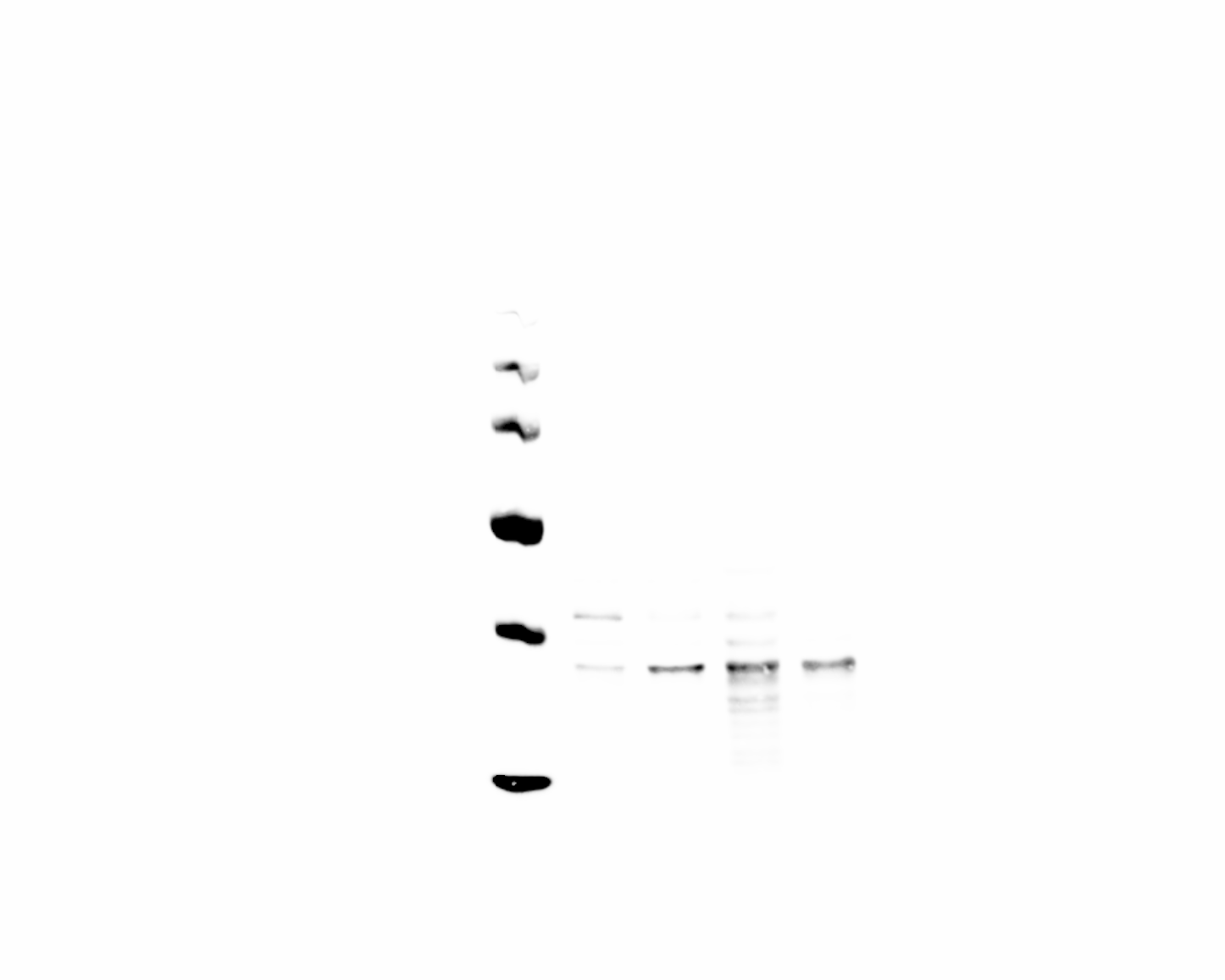

Supplement: Figure 3—figure supplement 1—source data 2. [file elife-95191-fig3-figsupp1-data2.zip › Figure 3-figure supplement 1 source data 2/rab27a.tif]

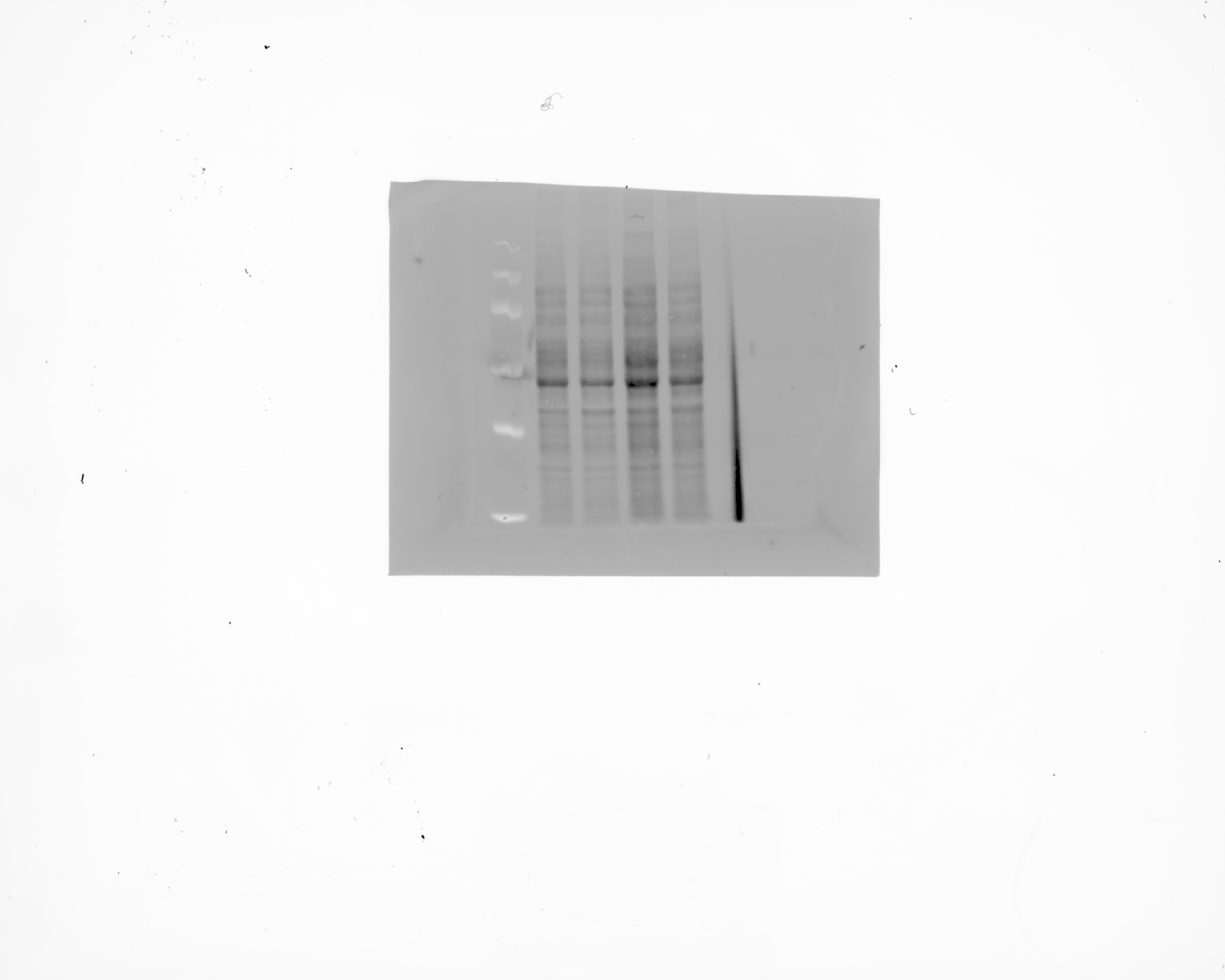

Supplement: Figure 3—figure supplement 1—source data 3. [file elife-95191-fig3-figsupp1-data3.zip › Figure 3-figure supplement 1 source data 3/Stain Free.tif]

Figure 3—figure supplement 1

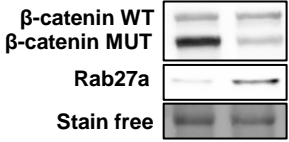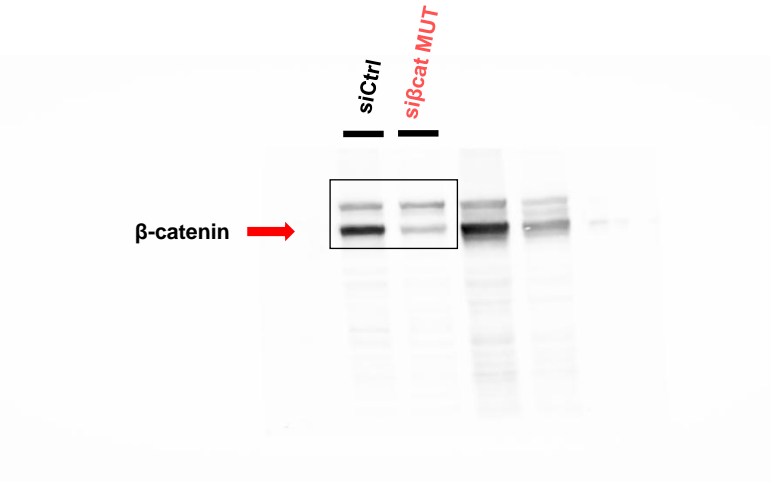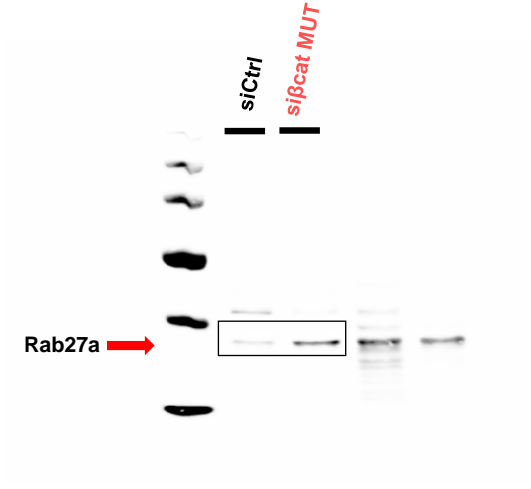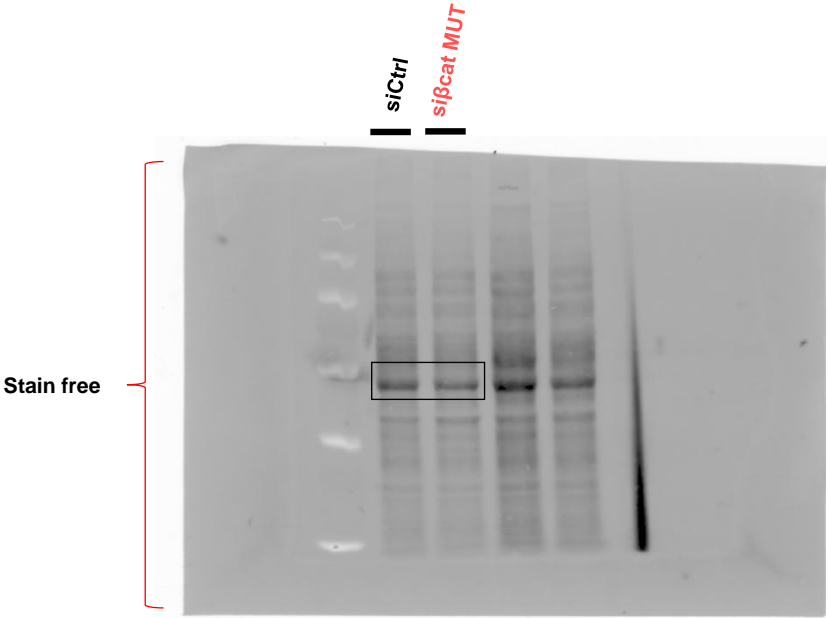

Supplement: Figure 3—figure supplement 1—source data 4. [file elife-95191-fig3-figsupp1-data4.pdf]

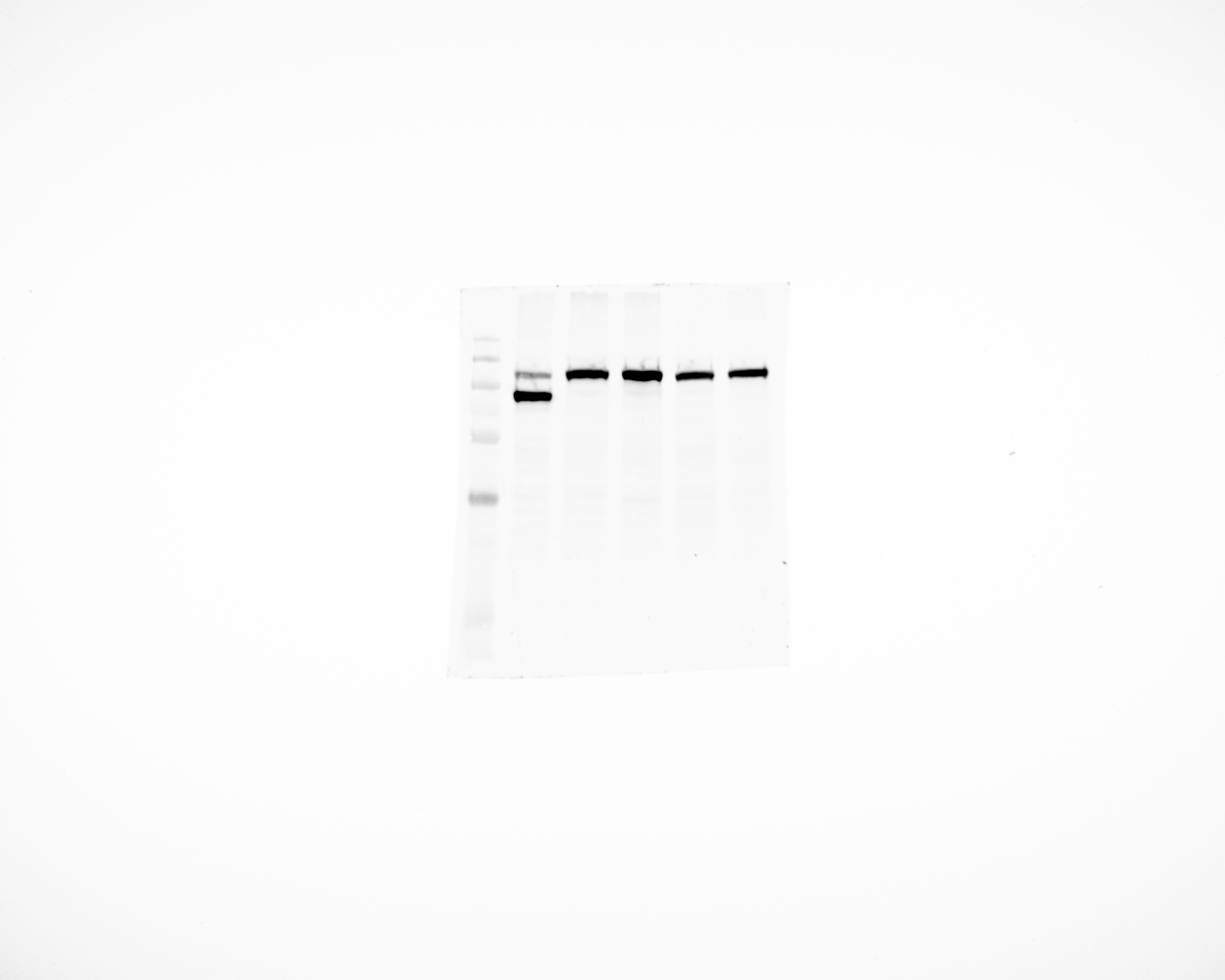

Supplement: Figure 3—figure supplement 3—source data 1. [file elife-95191-fig3-figsupp3-data1.zip › Figure 3-figure supplement 3 source data 1/b-catenin.tif]

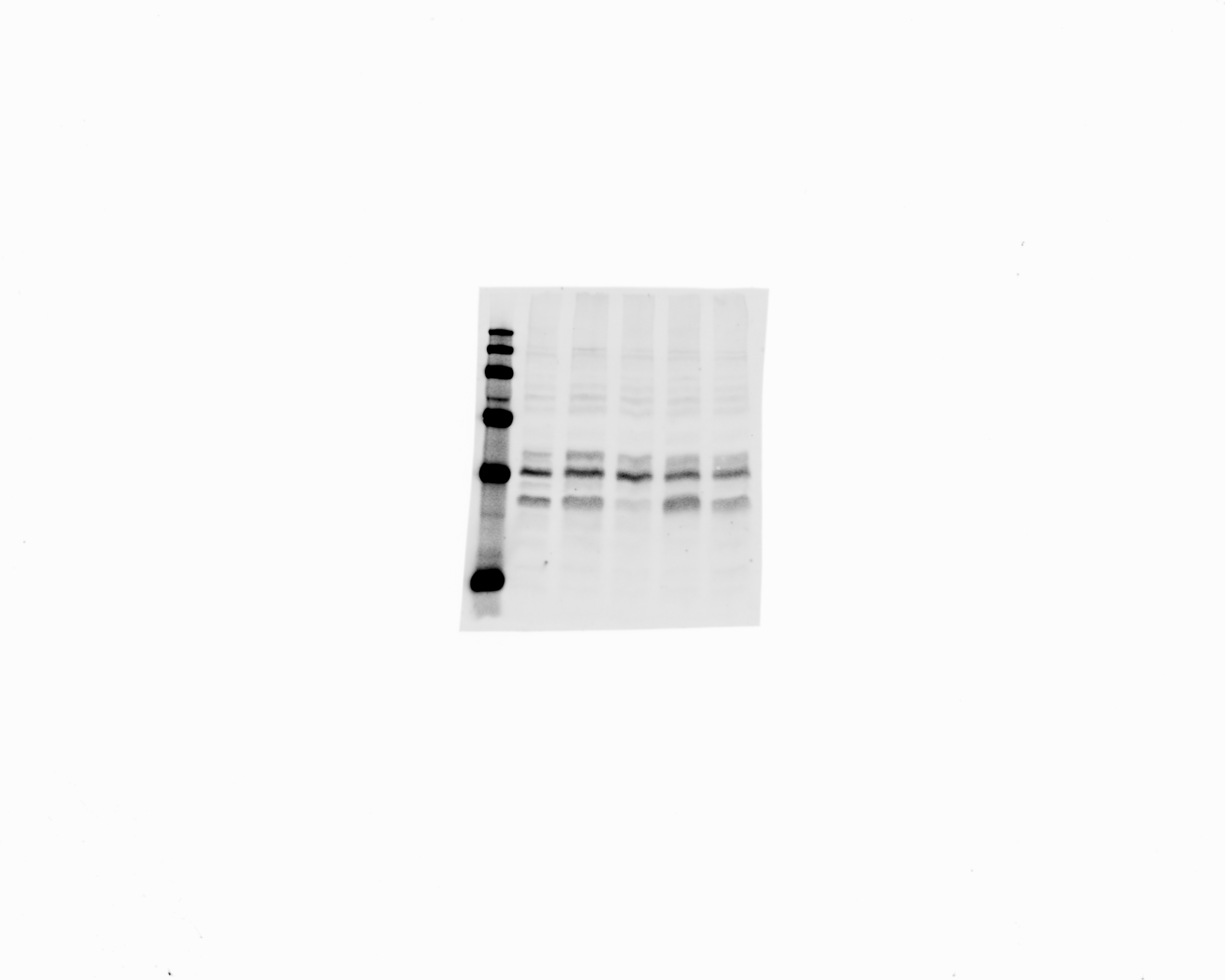

Supplement: Figure 3—figure supplement 3—source data 2. [file elife-95191-fig3-figsupp3-data2.zip › Figure 3-figure supplement 3 source data 2/rab27a.tif]

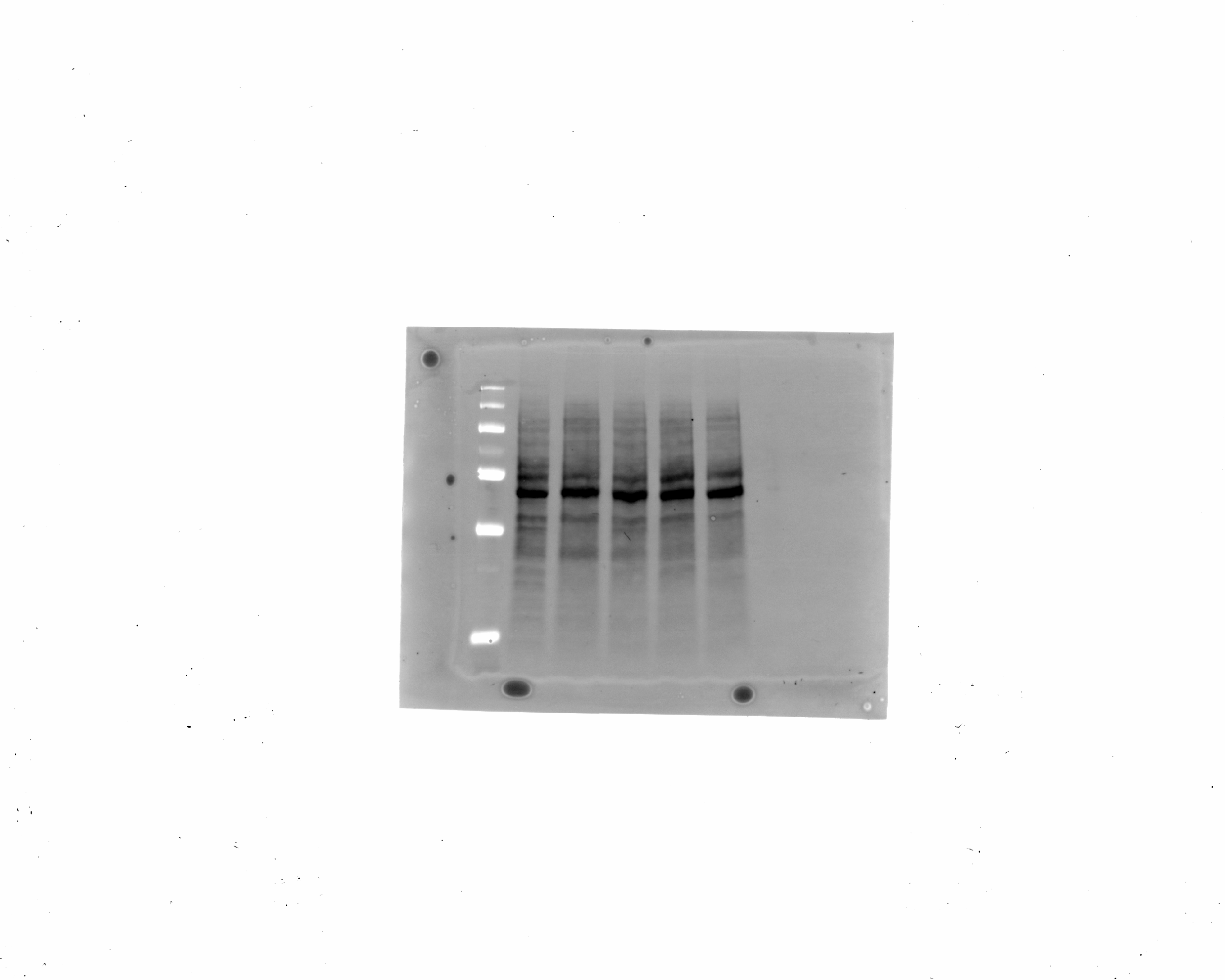

Supplement: Figure 3—figure supplement 3—source data 3. [file elife-95191-fig3-figsupp3-data3.zip › Figure 3-figure supplement 3 source data 3/Stain free.tif]

Figure 3—figure supplement 3

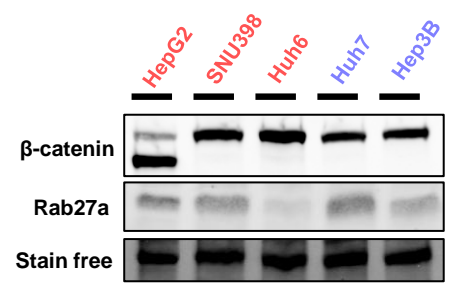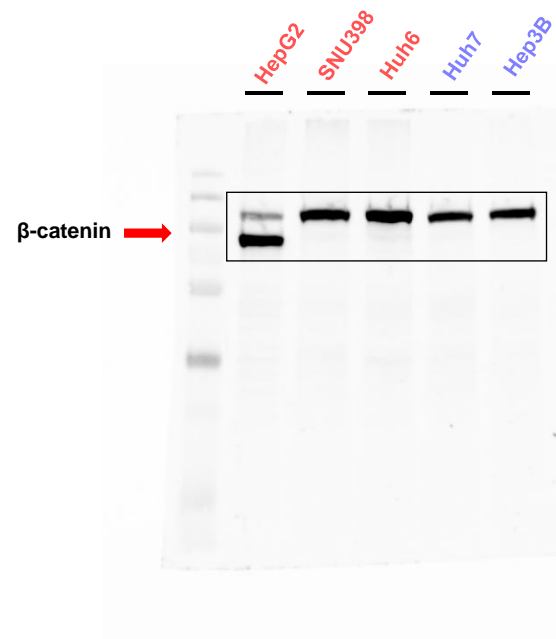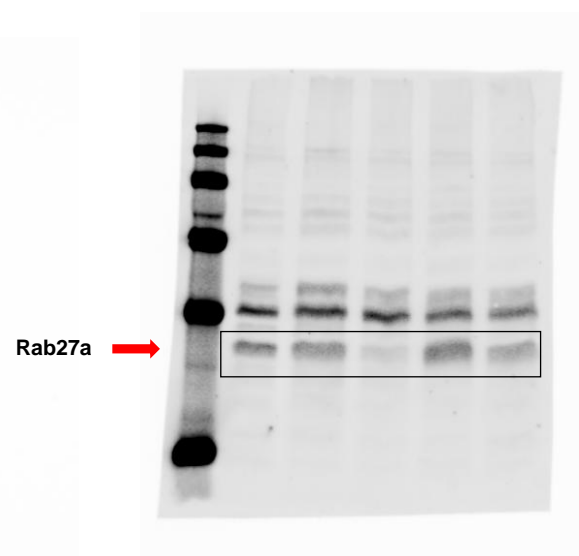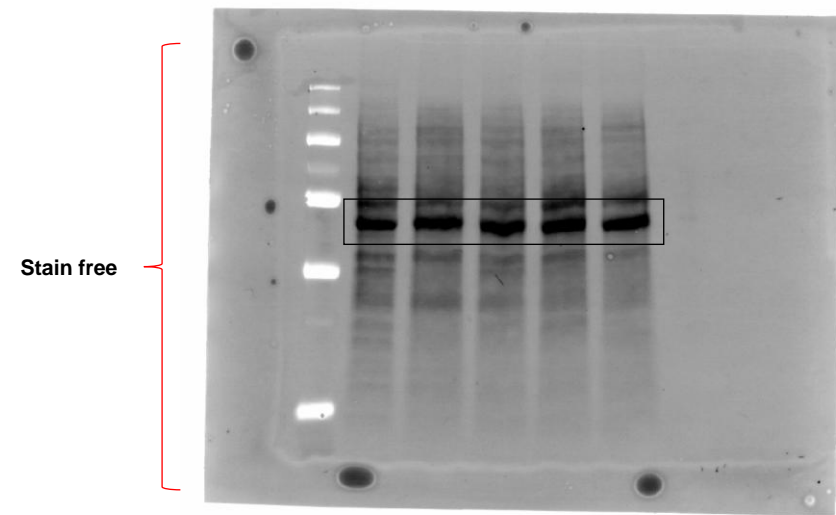

Supplement: Figure 3—figure supplement 3—source data 4. [file elife-95191-fig3-figsupp3-data4.pdf]

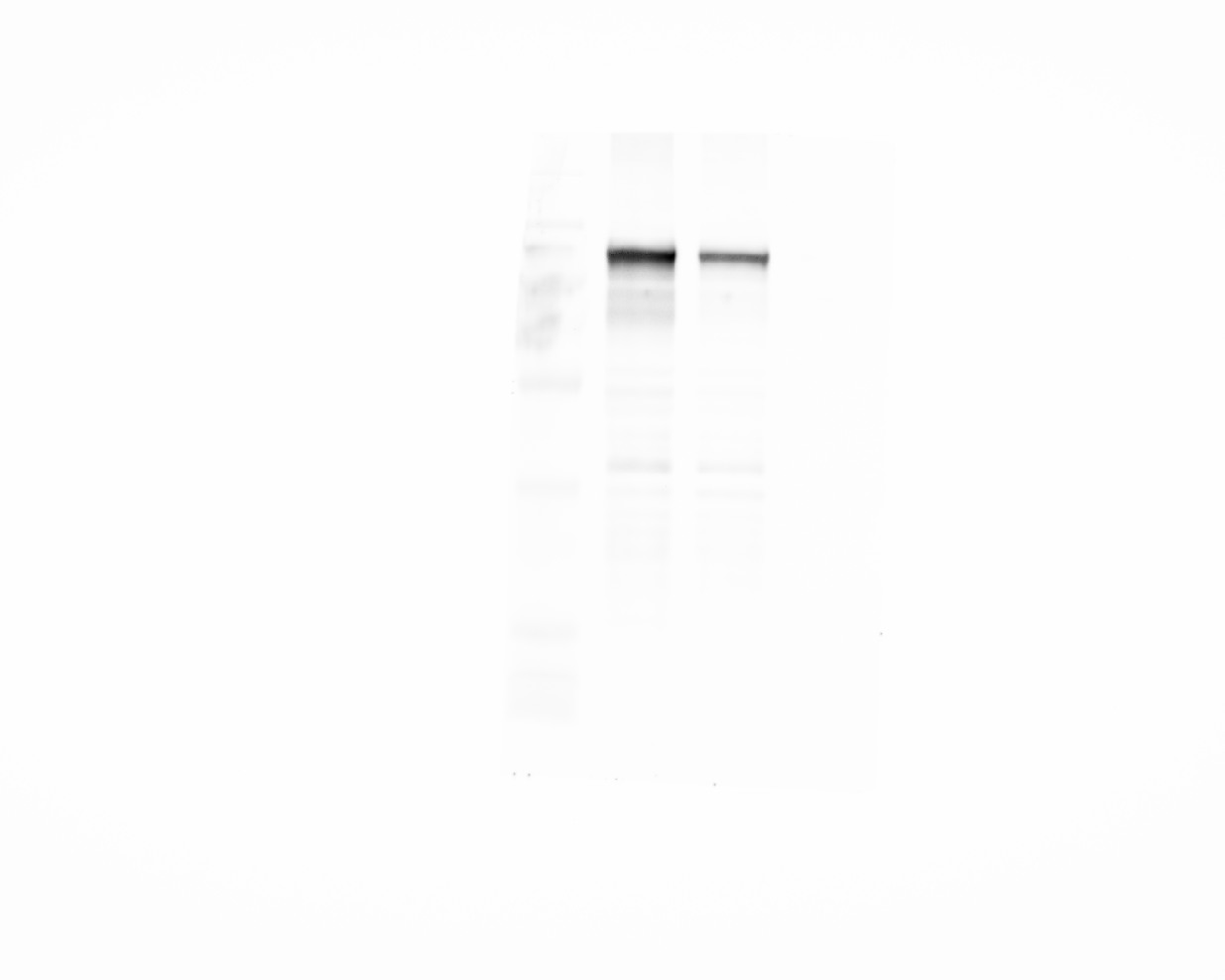

Supplement: Figure 3—figure supplement 4—source data 1. [file elife-95191-fig3-figsupp4-data1.zip › Figure 3-figure supplement 4 source data 1/b-catenin.tif]

Figure 3—figure supplement 4

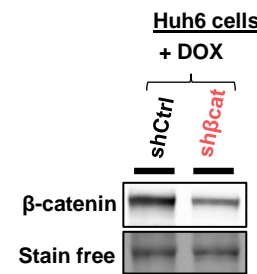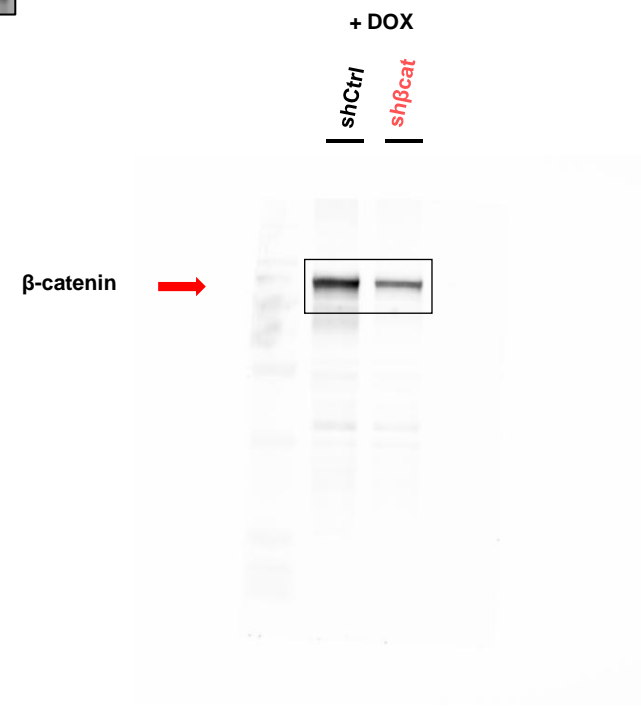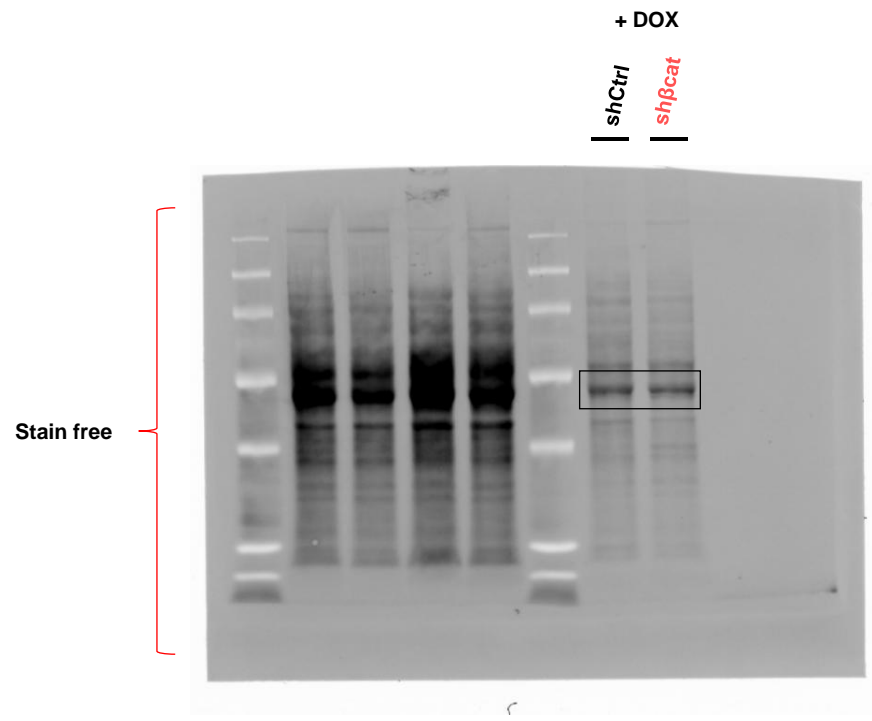

Supplement: Figure 3—figure supplement 4—source data 3. [file elife-95191-fig3-figsupp4-data3.pdf]

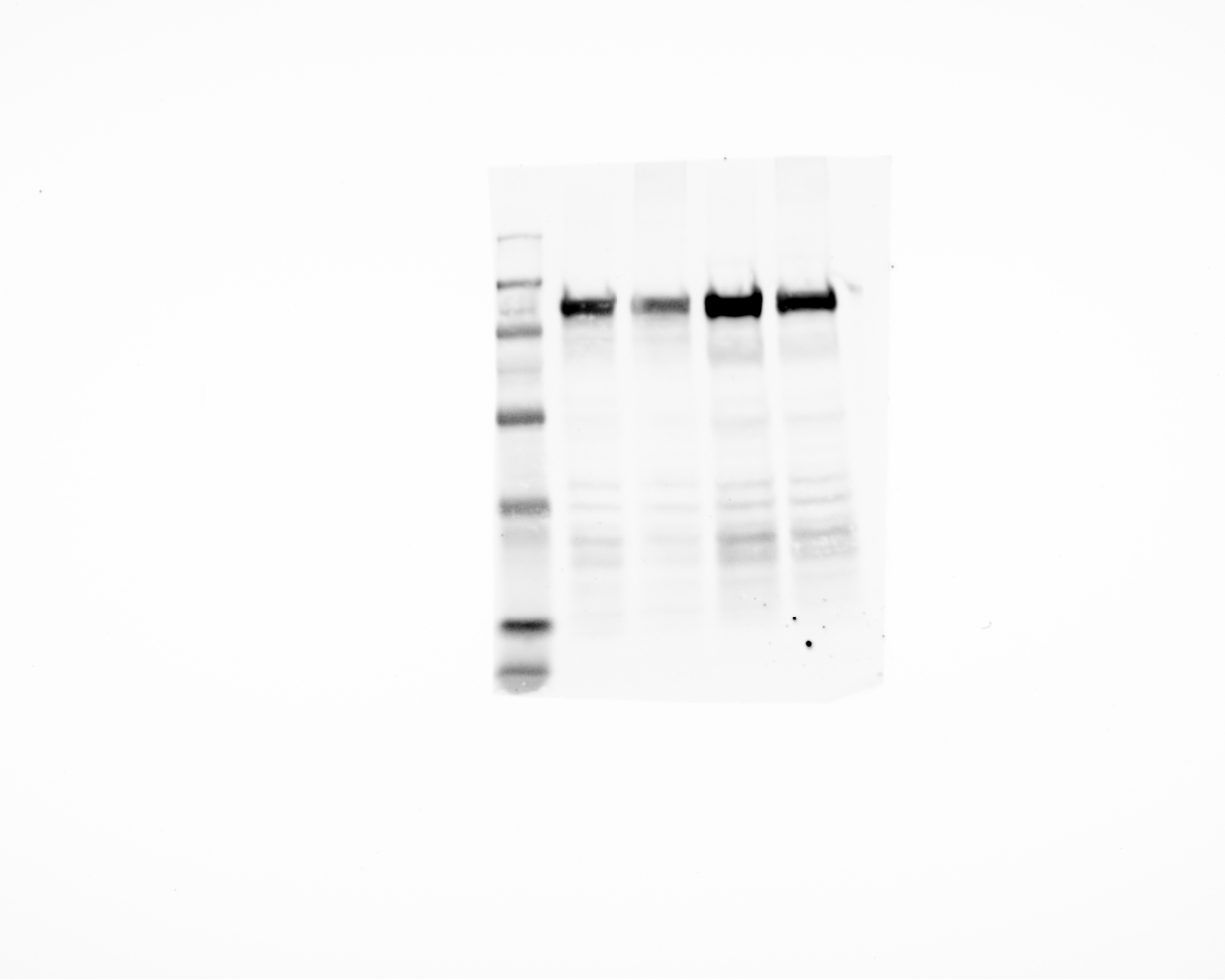

Supplement: Figure 3—figure supplement 4—source data 4. [file elife-95191-fig3-figsupp4-data4.zip › Figure 3-figure supplement 4 source data 4/b-catenin.tif]

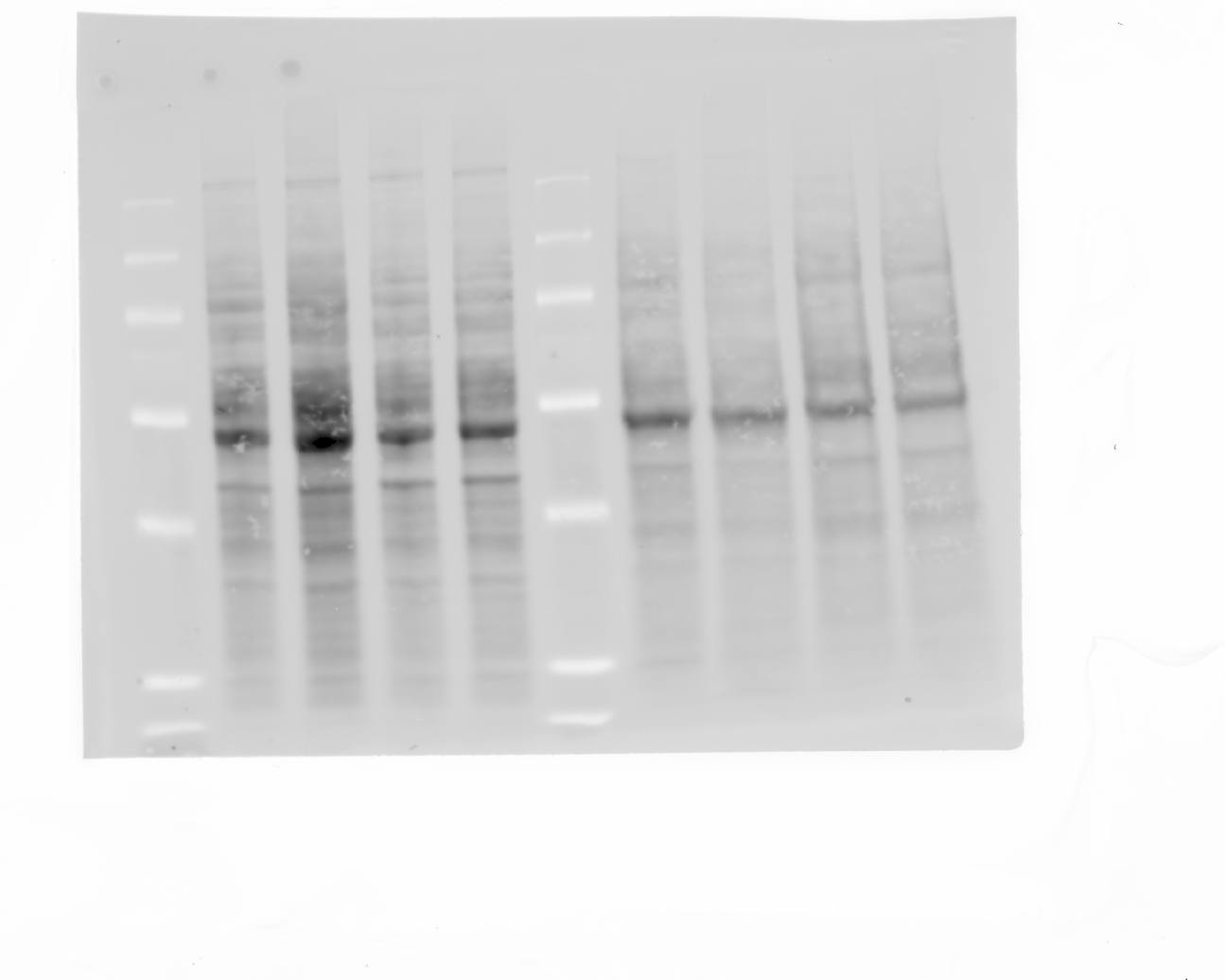

Supplement: Figure 3—figure supplement 4—source data 5. [file elife-95191-fig3-figsupp4-data5.zip › Figure 3-figure supplement 4 source data 5/Stain free.tif]

Figure 3—figure supplement 4

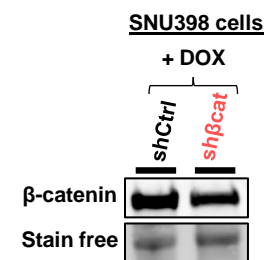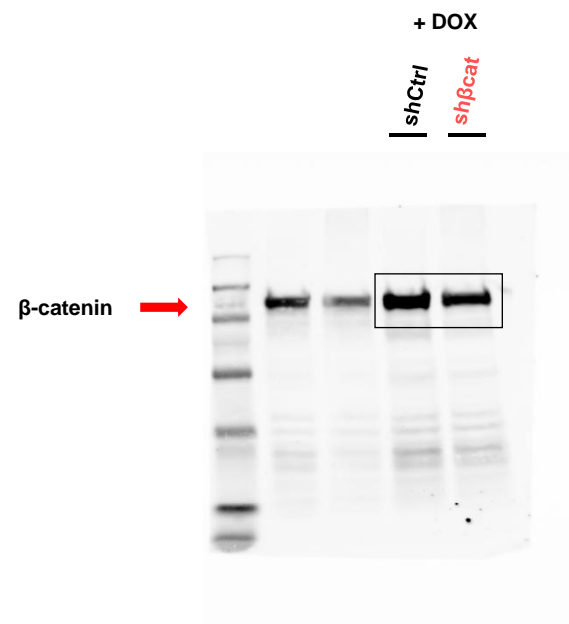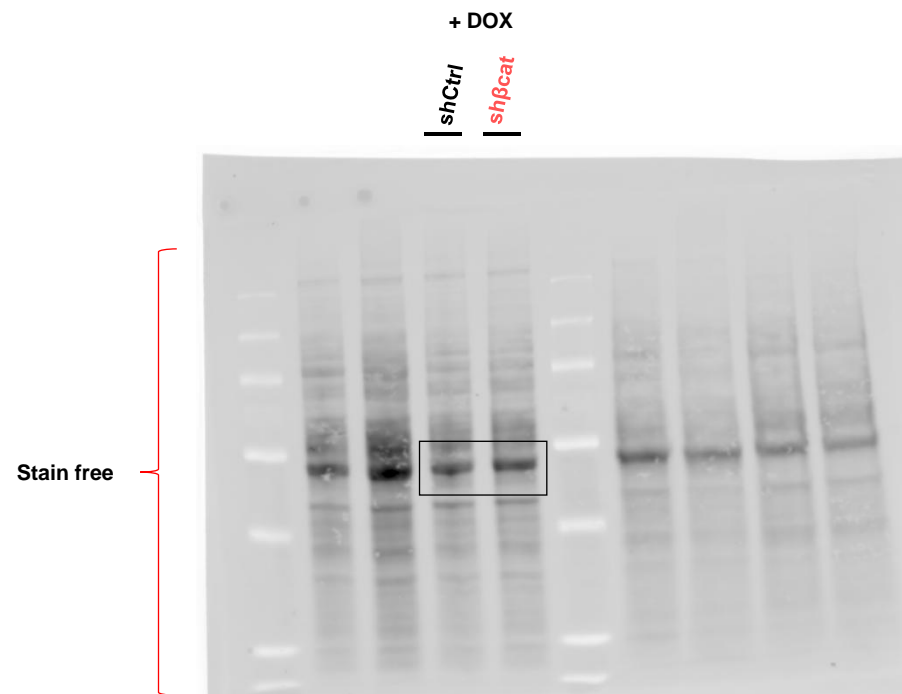

Supplement: Figure 3—figure supplement 4—source data 6. [file elife-95191-fig3-figsupp4-data6.pdf]

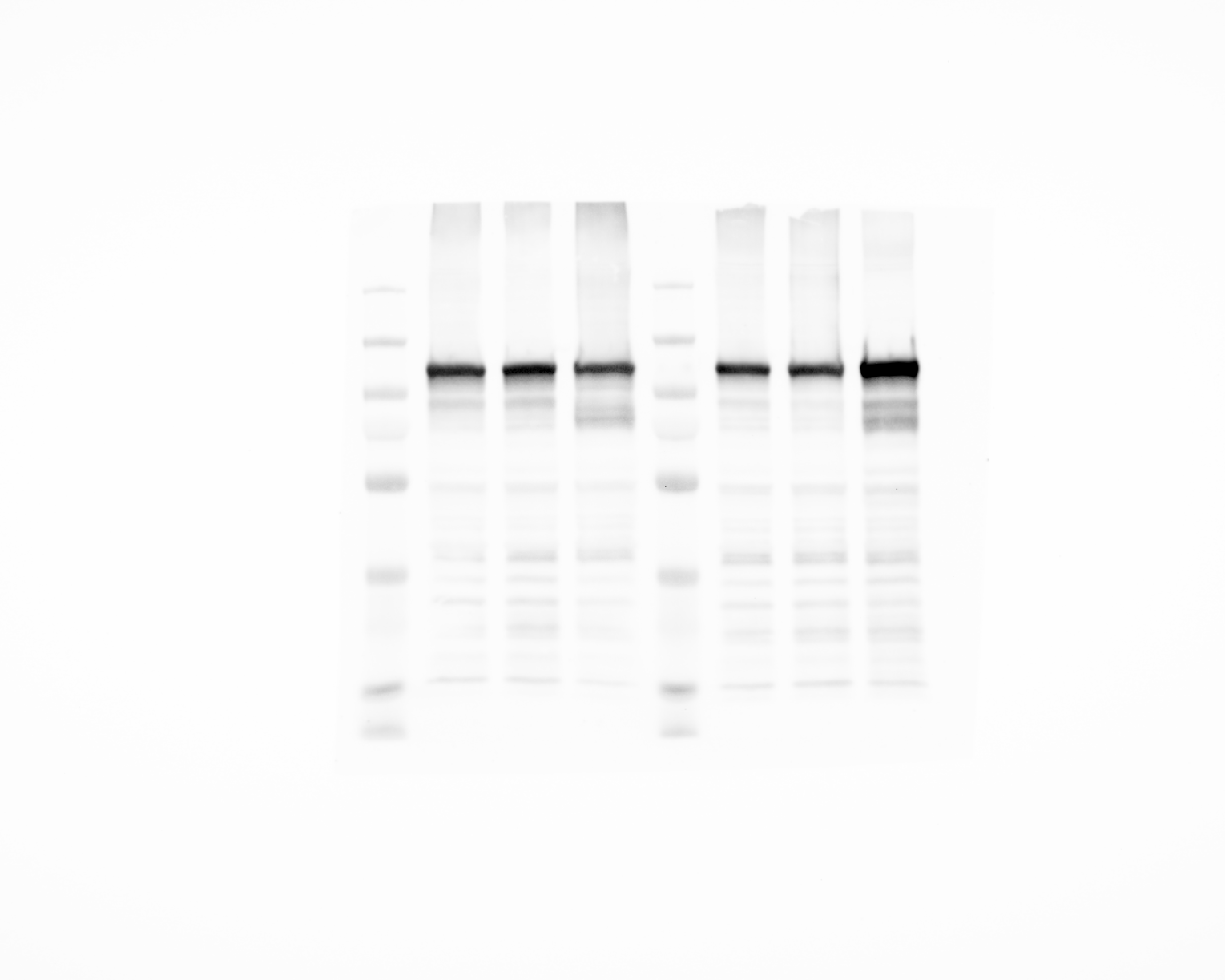

Supplement: Figure 4—figure supplement 1—source data 1. [file elife-95191-fig4-figsupp1-data1.zip › Figure 4-figure supplement 1 source data 1/b-catenin.tif]

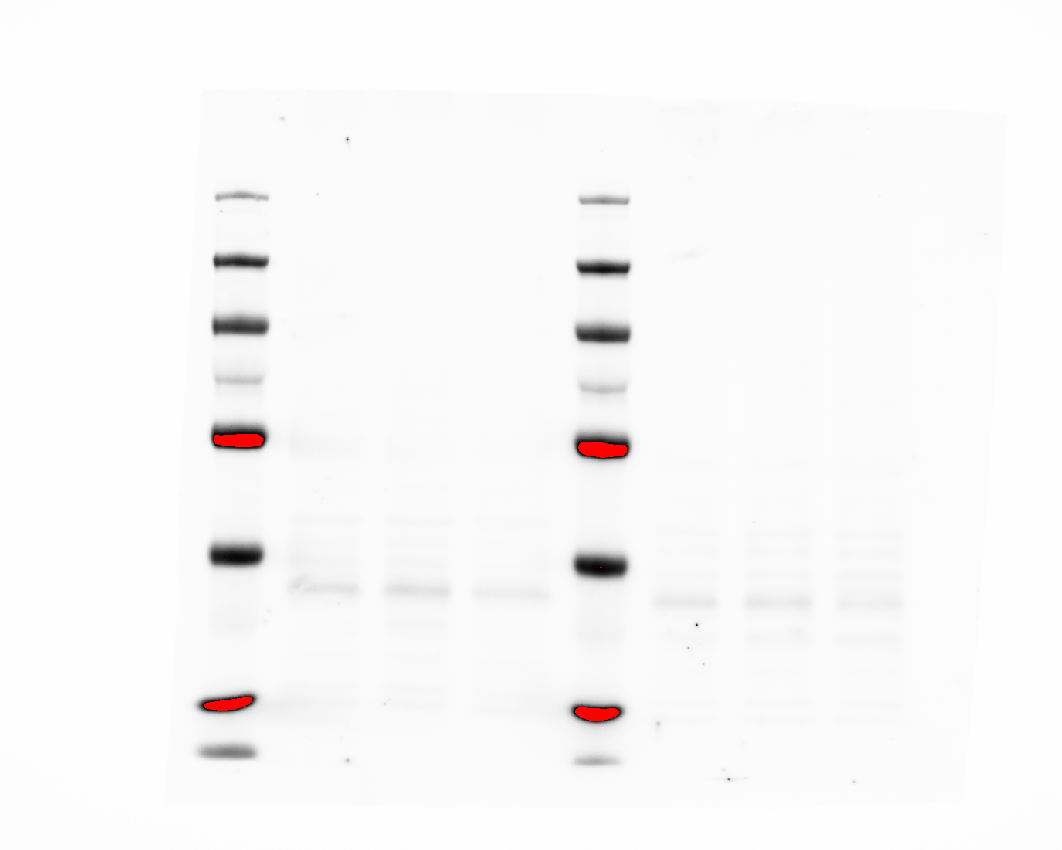

Supplement: Figure 4—figure supplement 1—source data 2. [file elife-95191-fig4-figsupp1-data2.zip › Figure 4-figure supplement 1 source data 2/rab27a.tif]

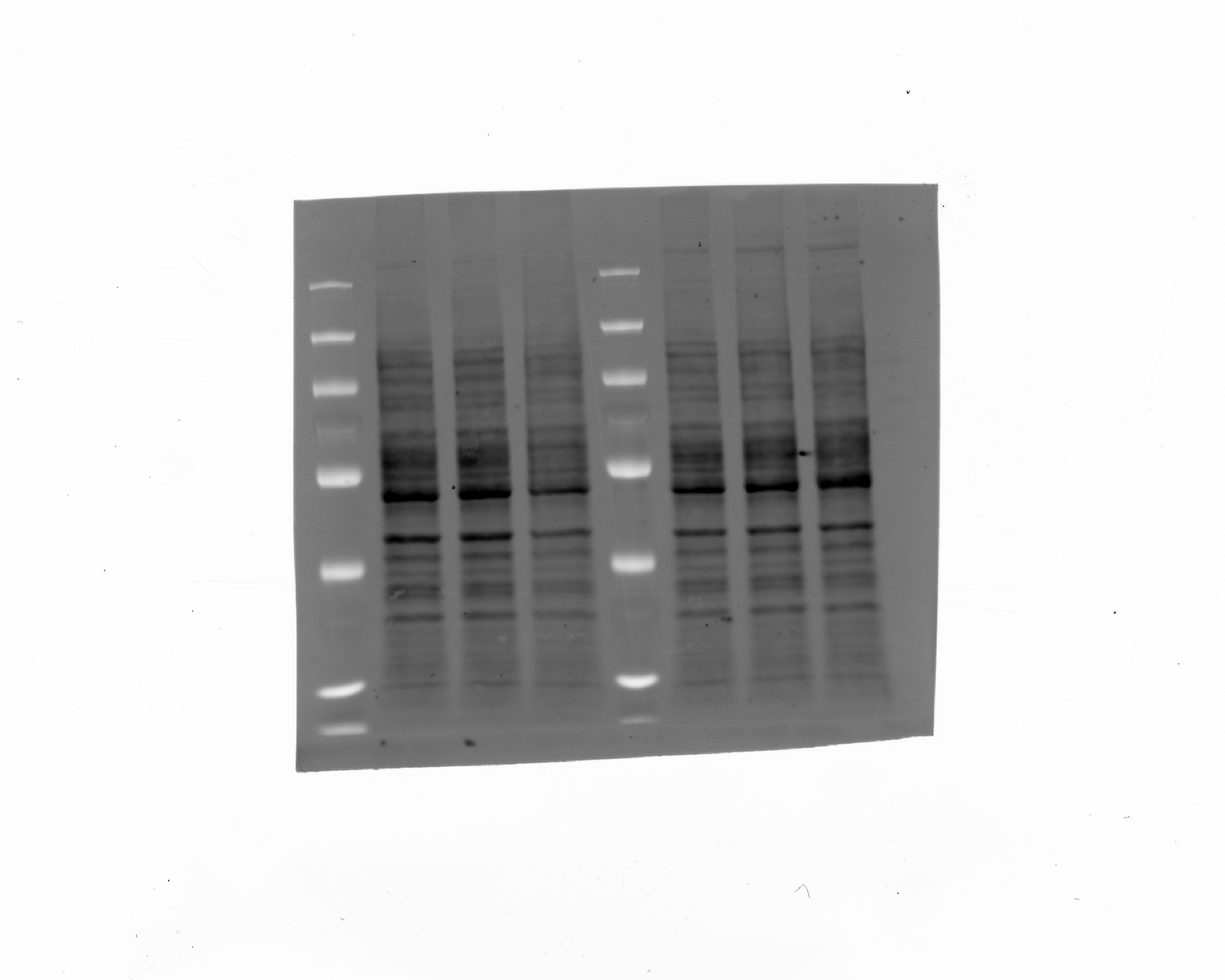

Supplement: Figure 4—figure supplement 1—source data 3. [file elife-95191-fig4-figsupp1-data3.zip › Figure 4-figure supplement 1 source data 3/Stain free.tif]

### Figure 4—figure supplement 1

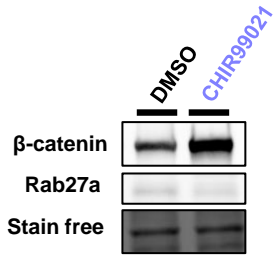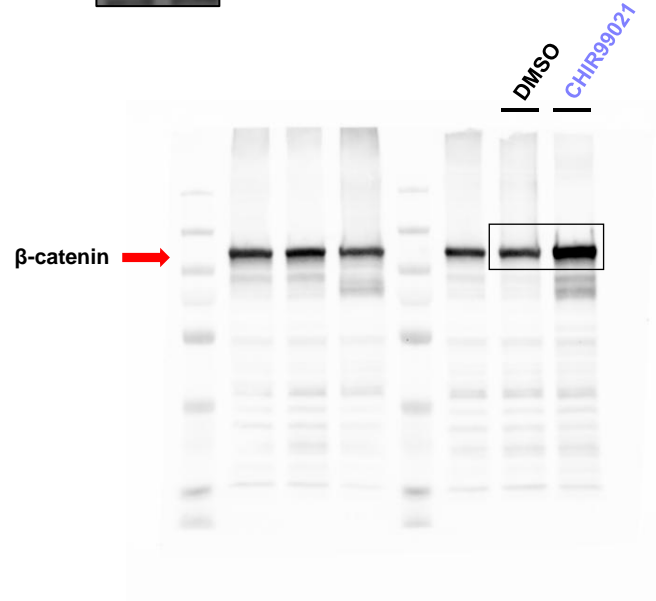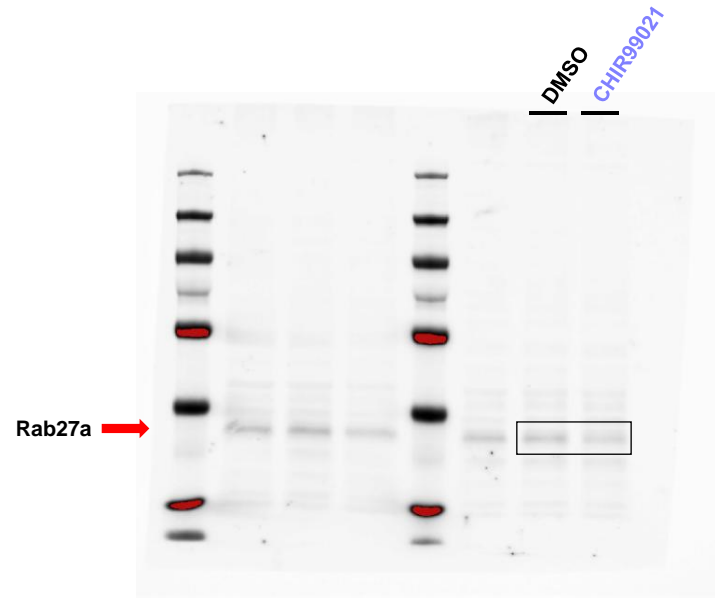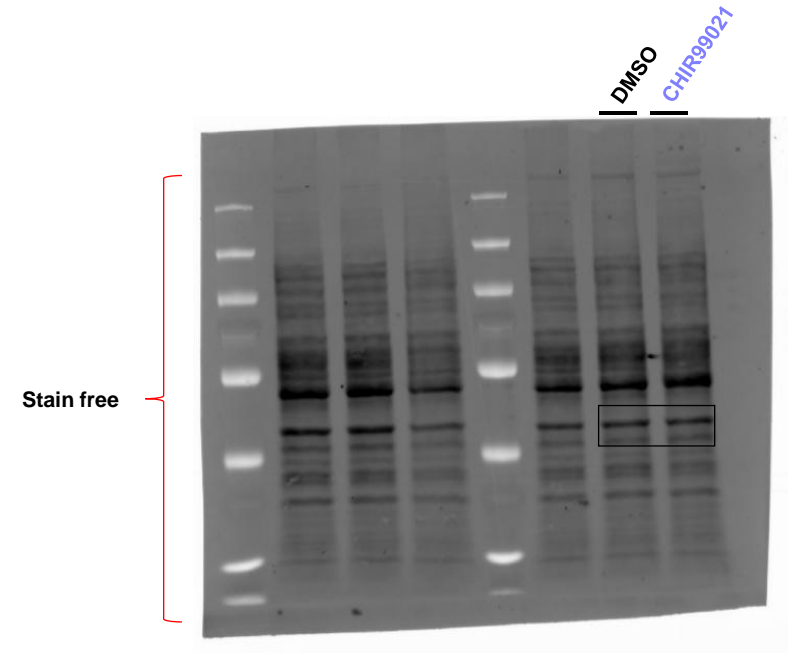

Supplement: Figure 4—figure supplement 1—source data 4. [file elife-95191-fig4-figsupp1-data4.pdf]

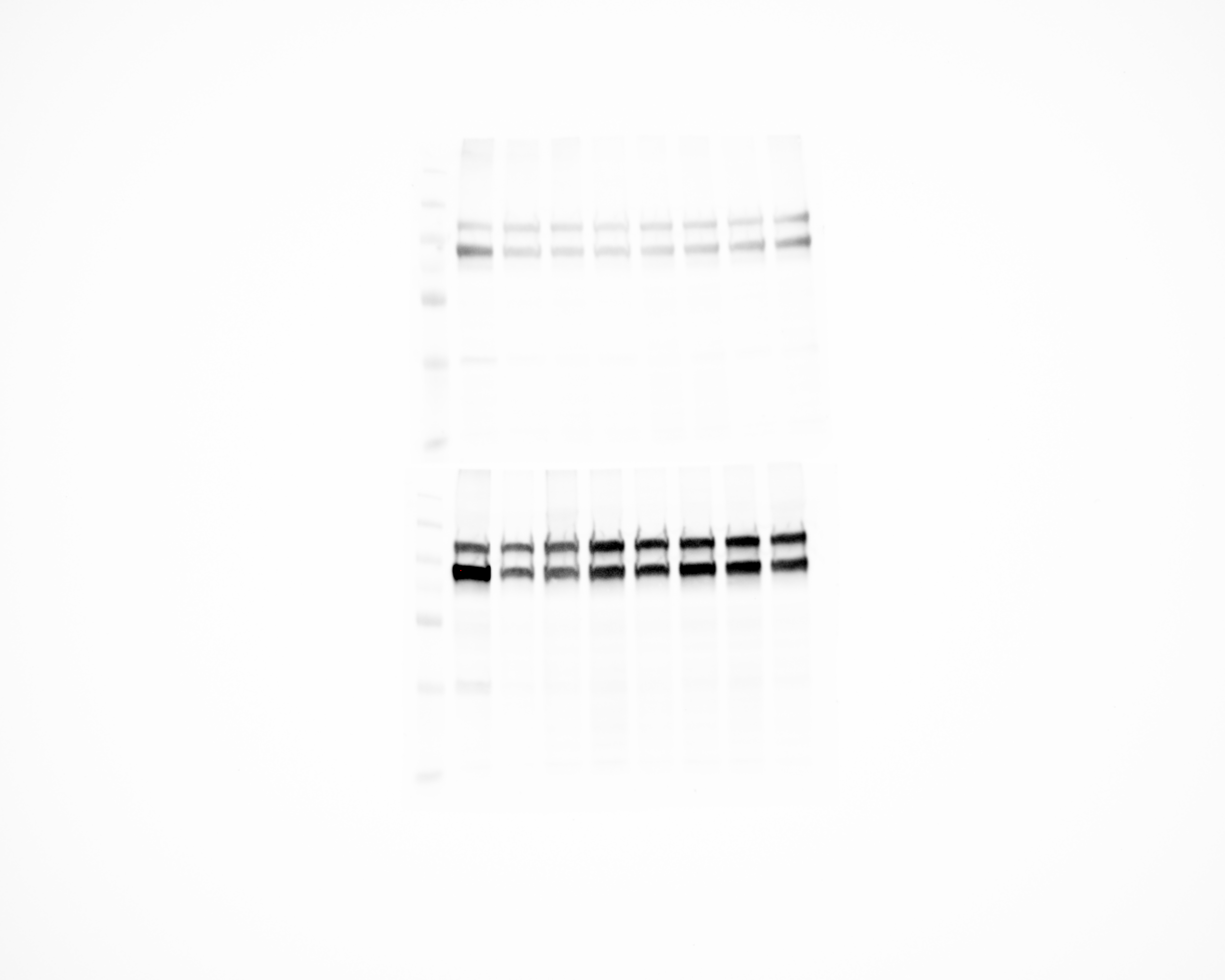

Supplement: Figure 4—figure supplement 2—source data 1. [file elife-95191-fig4-figsupp2-data1.zip › Figure 4-figure supplement 2 source data 1/b-catenin.tif]

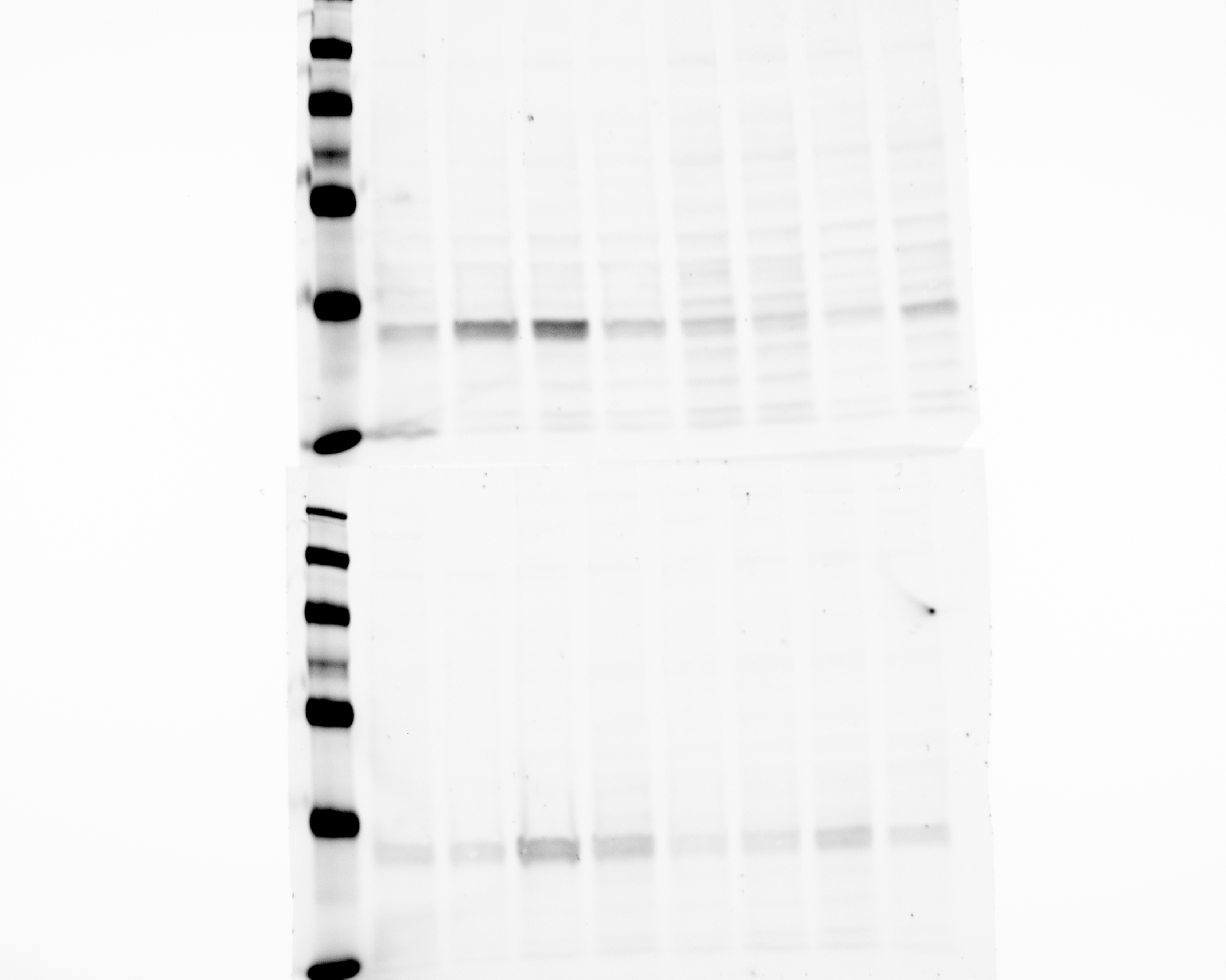

Supplement: Figure 4—figure supplement 2—source data 2. [file elife-95191-fig4-figsupp2-data2.zip › Figure 4-figure supplement 2 source data 2/rab27a.tif]

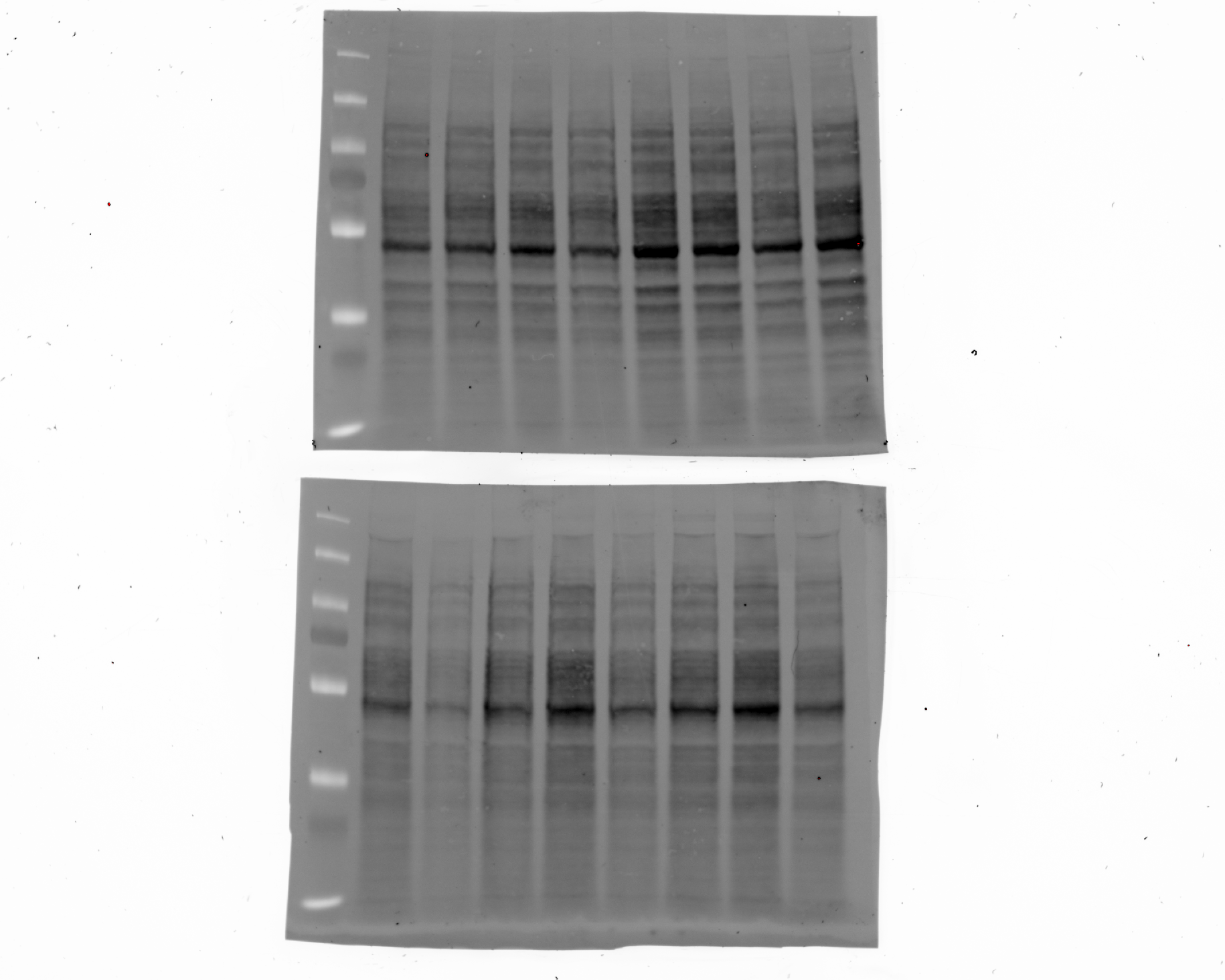

Supplement: Figure 4—figure supplement 2—source data 3. [file elife-95191-fig4-figsupp2-data3.zip › Figure 4-figure supplement 2 source data 3/Stain free.tif]

Figure 4—figure supplement 2

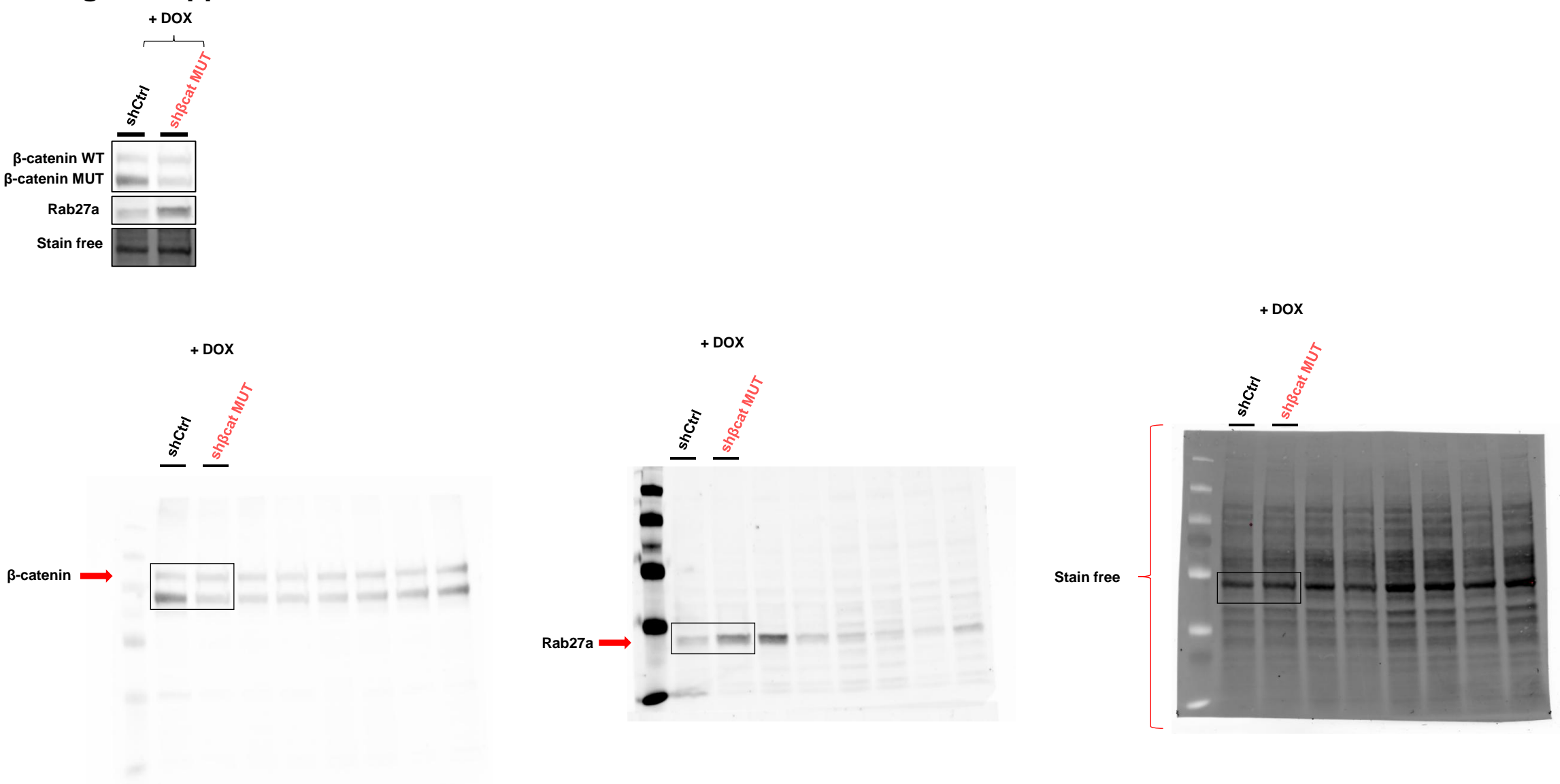

Supplement: Figure 4—figure supplement 2—source data 4. [file elife-95191-fig4-figsupp2-data4.pdf]

Figure 4—figure supplement 3

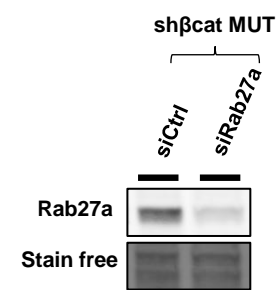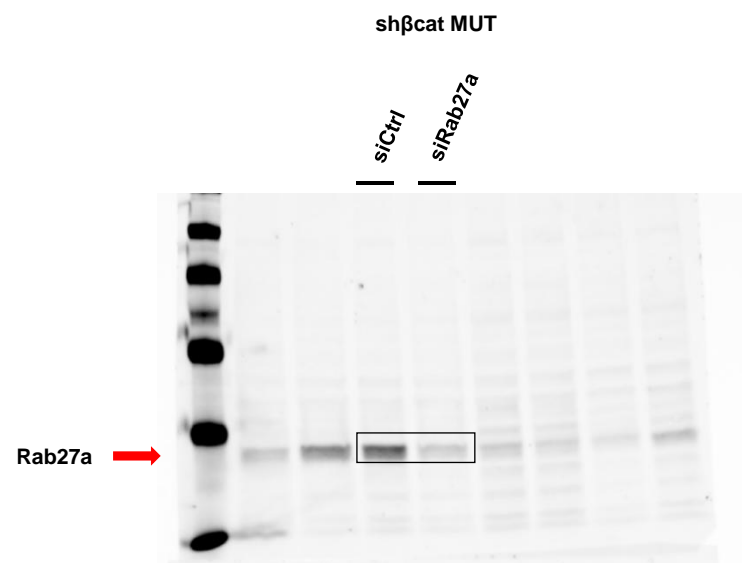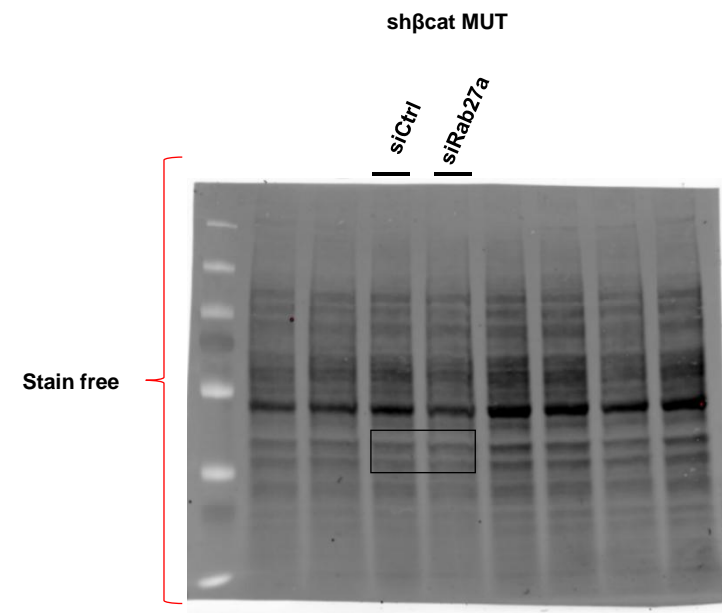

Supplement: Figure 4—figure supplement 3—source data 3. [file elife-95191-fig4-figsupp3-data3.pdf]
